# Impact of Exercise and Aging on Mitochondrial Homeostasis in Skeletal Muscle: Roles of ROS and Epigenetics

**DOI:** 10.3390/cells11132086

**Published:** 2022-06-30

**Authors:** Jialin Li, Zhe Wang, Can Li, Yu Song, Yan Wang, Hai Bo, Yong Zhang

**Affiliations:** 1 Tianjin Key Laboratory of Exercise Physiology and Sports Medicine, Institute of Exercise and Health, Tianjin University of Sport, Tianjin 301617, China; lijialin_mito@yeah.net (J.L.); mito_sirwang@163.com (Z.W.); canli_mito@sina.com (C.L.); songyu@tjus.edu.cn (Y.S.); haiyaojing1208@126.com (Y.W.); 2 Department of Military Training Medicines, Logistics University of Chinese People’s Armed Police Force, Tianjin 300162, China

**Keywords:** aging, exercise, ROS, mitochondrial, epigenetics, skeletal muscle

## Abstract

Aging causes degenerative changes such as epigenetic changes and mitochondrial dysfunction in skeletal muscle. Exercise can upregulate muscle mitochondrial homeostasis and enhance antioxidant capacity and represents an effective treatment to prevent muscle aging. Epigenetic changes such as DNA methylation, histone posttranslational modifications, and microRNA expression are involved in the regulation of exercise-induced adaptive changes in muscle mitochondria. Reactive oxygen species (ROS) play an important role in signaling molecules in exercise-induced muscle mitochondrial health benefits, and strong evidence emphasizes that exercise-induced ROS can regulate gene expression via epigenetic mechanisms. The majority of mitochondrial proteins are imported into mitochondria from the cytosol, so mitochondrial homeostasis is regulated by nuclear epigenetic mechanisms. Exercise can reverse aging-induced changes in myokine expression by modulating epigenetic mechanisms. In this review, we provide an overview of the role of exercise-generated ROS in the regulation of mitochondrial homeostasis mediated by epigenetic mechanisms. In addition, the potential epigenetic mechanisms involved in exercise-induced myokine expression are reviewed.

## 1. Introduction

Skeletal muscle aging leads to oxidative stress and mitochondrial dysfunction [1]. The mitochondrial quality control system in aging skeletal muscle is dysregulated, including reduced mitochondrial biogenesis, an imbalance of mitochondrial dynamics, and impaired mitophagy [2,3,4]. Therefore, it is important to maintain the balance of the mitochondrial quality control system in aging skeletal muscle.

Exercise is an effective nondrug intervention to improve the health of aging skeletal muscle, but the underlying mechanism of exercise-induced mitochondrial adaptation in skeletal muscle remains unclear. Recent studies have demonstrated that exercise can play a role in promoting mitochondrial health in skeletal muscle by upregulating mitochondrial biogenesis, improving mitochondrial dynamics balance, and promoting mitophagy [5,6].

Epigenetic mechanisms can integrate environmental factors to regulate gene expression. With increasing age, the alterations of epigenetic markers of skeletal muscle mitochondria-related genes could induce mitochondrial dysfunction, resulting in decreased skeletal muscle function [7,8,9,10,11]. Exercise ameliorates aging-induced mitochondrial dysfunction in skeletal muscle [12,13]. Exercise acts as a powerful stimulus that induces changes in a wide range of epigenetic markers. Recent evidence suggests that exercise-induced reactive oxygen species (ROS) act as epigenetic modulators to regulate skeletal muscle mitochondria-related gene epigenetic modifications and protein expression in a direct and/or indirect manner, thus maintaining mitochondrial homeostasis and promoting skeletal muscle health [14,15].

Skeletal muscle can secrete a variety of myokines, which promote crosstalk between skeletal muscle and other organs. Recent evidence suggests that the expression of myokines is also regulated by epigenetic modifications [16,17,18,19,20,21].

In this paper, we review the current findings on the regulation of mitochondrial homeostasis in skeletal muscle by aging and exercise, especially the role of epigenetic modifications in the regulatory mechanisms of mitochondrial homeostasis. We specifically focused on the role of ROS in the regulation of epigenetic modifications. Finally, we also describe the important role of exercise-induced alterations in epigenetic modifications in regulating myokine expression.

## 2. Aging-Induced Mitochondrial Dysfunction in Skeletal Muscle

In mammals, skeletal muscle is composed of different types of muscle fibers. Rodent skeletal muscle consists of type I, IIa, IId/x, and IIb fibers, whereas human skeletal muscle consists of type I, IIa, and IId/x fibers [22,23]. Type I slow-twitch oxidative fibers contract slowly and are rich in mitochondria, type IIa fibers contract quickly and are rich in mitochondria, and type IId/x fibers and type IIb fibers contract quickly and contain fewer mitochondria [24]. Differences in the distribution of skeletal muscle fiber types are noted between males and females. The female vastus lateralis has more type I muscle fibers and fewer type IIa and IId/x muscle fibers. This difference is also reflected in results from rats and mice [25,26]. Daniela D’Amico et al. [27] demonstrated that female mice have a higher number of type I muscle fibers in the soleus muscle and a higher number of type IIa fibers in the EDL muscle than male mice. Mitochondria provide energy for skeletal muscle contraction through oxidative phosphorylation (OXPHOS). Mitochondria are also crucial for skeletal muscle cell redox regulation and cell viability. As early as in 2005, scientists described mitochondria as multifunctional signaling centers that associate cellular function with metabolism and age [28]. Mitochondria play critical roles in cellular homeostasis in response to intracellular and extracellular stress, epigenetics, and aging. Mitochondrial dysfunction is a hallmark of aging [29]. Skeletal muscle mitochondrial function changes with age, including decreased mitochondrial membrane potential, increased ROS production, altered mitochondrial enzyme activity, and decreased mitochondrial adenosine triphosphate (ATP) synthesis capacity [2]. Therefore, mitochondria-targeted regulation is considered to be one of the factors improving skeletal muscle cell function. Some evidence indicates that aging can be delayed by targeted regulation of mitochondrial function [30].

### 2.1. Aging-Associated Reductions in Mitochondrial Biogenesis

The mitochondrial content of skeletal muscle decreases with age and is mainly manifested as a decrease in mitochondrial number and density and a decrease in mitochondrial deoxyribonucleic acid (DNA) copy number and mitochondrial protein expression [2]. Peroxisome proliferator-activated receptor γ coactivator-1α (PGC-1α) is an important gene regulating mitochondrial biogenesis and skeletal muscle insulin sensitivity. PGC-1α is decreased in the skeletal muscle of aged mice. Overexpression of PGC-1α in the skeletal muscle of aging mice resulted in increased skeletal muscle mitochondrial content, a new balance of myosin heavy chain isoforms, increased levels of mitophagy markers, and reduced levels of proteasome markers, and these changes were similar to the molecular features of young mouse skeletal muscle [31]. Yang et al. [32] demonstrated that PGC-1α overexpression in skeletal muscle of 24-month-old male mice decreased muscle fatigue and prevented sarcopenia. Therefore, promoting the regulation of mitochondrial biogenesis may represent a potential target for preventing skeletal muscle aging.

### 2.2. Aging-Associated Alterations in Mitochondrial Dynamics

Mitochondrial dynamics are also important factors in maintaining mitochondrial function. Imbalances in mitochondrial dynamics and abnormal mitophagy increase with age [3]. Faitg et al. [33] demonstrated that dynamin-related protein 1 (DRP1) content was significantly elevated in the soleus and gastrocnemius muscles of aging rats. Touvier et al. [34] demonstrated that specific overexpression of DRP1 in skeletal muscle revealed mitochondrial network reorganization and reduced mitochondrial DNA copy number and that activation of the dsRNA-dependent protein kinase/eukaryotic initiation factor 2/fibroblast growth factor 21 pathway by Drp1 overexpression resulted in diminished skeletal muscle protein synthesis and downregulation of the growth hormone pathway. Muscle-specific knockout of Drp1 in mice resulted in a severe myopathic phenotype, including muscle atrophy, weakness, upregulation of forkhead box O3 (FOXO3) expression and downregulation of atrophy-related ubiquitin ligases, such as muscle atrophy F-box and muscle RING finger 1 (MuRF-1) expression [35]. In addition, Sebastián et al. [36] found that mitofusin 2 (Mfn2) expression was decreased in the skeletal muscle of aging mice and that Mfn2 knockout in mouse skeletal muscle produces aging-related features, such as reduced mitophagy and decreased mitochondrial function. The aging-induced decrease in Mfn2 expression underlies age-related changes in skeletal muscle metabolic disorders and sarcopenia. Tezze et al. [37] demonstrated that optic atrophy 1 (OPA1) is significantly reduced in the skeletal muscle of aging mice and that specific knockout of OPA1 in skeletal muscle leads to inhibition of protein synthesis, promotion of protein degradation, and induction of atrophy-related gene expression, resulting in a precocious aging phenotype in mice. Furthermore, imbalances in mitochondrial dynamics in aging skeletal muscle may lead to the appearance of giant mitochondria [38,39]. Navratila et al. [40] demonstrated that the appearance of giant mitochondria is associated with a decrease in OPA1 in aging skeletal muscle independent of the MFN2 content. Exercise may regulate the balance of skeletal muscle mitochondrial dynamics through ROS, inhibiting mitochondrial swelling and giant mitochondria [41,42,43]. Therefore, targeted regulation of mitochondrial dynamics may represent a potential target for the prevention of skeletal muscle aging.

### 2.3. Aging-Associated Alterations in Mitophagy

With increasing age, mitophagy is impaired, and dysfunctional mitochondria accumulate in mouse skeletal muscle [4,44,45]. In aging skeletal muscle, a decrease in mitophagy markers is closely related to mitochondrial quality and skeletal muscle function. Leduc-Gaudet et al. [46] demonstrated that the Parkin content was significantly reduced in the skeletal muscle of aging mice. Overexpression of Parkin in mice counteracts age-related sarcopenia, upregulates the protein kinase B (Akt)–mammalian target of rapamycin (mTOR) 1 pathway, promotes protein synthesis, inhibits protein degradation, and triggers skeletal muscle hypertrophy. Knockout of Parkin in mice resulted in decreased muscle strength and decreased mitochondrial respiratory function [47]. Upregulation of mitophagy using urolithiasis A could improve mitochondrial respiratory capacity, prolong lifespan in *Caenorhabditis elegans* (*C. elegans*), and attenuate age-related skeletal muscle dysfunction in rodents [48]. Improving mitophagy in aging skeletal muscle leads to increased mitochondrial biogenesis, improved mitochondrial function, and enhanced skeletal muscle health [4]. Therefore, the regulation of mitophagy in skeletal muscle is also a potential target for preventing skeletal muscle aging.

### 2.4. Aging-Associated Reductions in UPRmt

Mitochondrial unfolded protein response (UPRmt) is a highly conserved mitochondrial stress response. Originally discovered in mammalian cells and further characterized in *C. elegans*, UPRmt plays a regulatory role during aging [49,50]. As the degree of aging increases, the mechanism of UPRmt activation is inhibited [21,51]. Cordeiro et al. [52] demonstrated that the expression of UPRmt markers in skeletal muscle of 60- to 70-year-old males was positively correlated with the expression of various mitochondrial metabolism-related genes and exercise. The mRNA levels of the UPRmt-related genes activating transcription factor (ATF) 4 and caseinolytic protease (ClpP) were significantly reduced in the skeletal muscle of aging mice, whereas exercise activated UPRmt by upregulating the expression of these UPRmt-related genes [52,53]. In addition, Zhang et al. [54] demonstrated that the protein expression of the UPRmt marker genes HSP60, HSP10, and CLpP was decreased and that UPRmt was inhibited in muscle stem cells of aging mice. Multiple studies have shown that lifespan can be extended by activating UPRmt in *C. elegans*, *Drosophila*, and mice [51,55,56,57,58]. In conclusion, UPRmt is inhibited with increasing age, and exercise maintains mitochondrial protein homeostasis by activating UPRmt in aging skeletal muscle, which subsequently delays aging or prolongs lifespan.

### 2.5. Aging- and Exercise-Associated Alterations in MAMs

At present, the crosstalk between organelles has become an important topic, especially signal transduction between mitochondria and the endoplasmic reticulum (ER), which is an important factor in determining cell fate. Mitochondria-associated endoplasmic reticulum membranes (MAMs) play a crucial role in the crosstalk between mitochondria and the ER [59]. MAMs are regions of 10–25 nm wide juxtaposition of the ER membrane and mitochondria tethered by proteins without complete fusion or loss of organelle identity [60]. MAMs are important intracellular signaling hubs that play important roles in calcium (Ca^2+^) homeostasis regulation, oxidative stress, management of unfolded proteins, and coordination of the mitochondrial–ER function [61,62]. Ca^2+^ is an important signaling molecule in skeletal muscle that plays a key role in excitation and contraction coupling of skeletal muscle fibers, mitochondrial metabolism, and maintenance of cell survival [35,63,64]. The ER is the major intracellular Ca^2+^-storing organelle, and Ca^2+^ accumulation in mitochondria is largely dependent on the ER. MAMs allow rapid transfer of Ca^2+^ between two intracellular organelles [65]. The Ca^2+^ concentration in mitochondria is important in regulating the ATP production process. Mitochondrial Ca^2+^ accumulation stimulates aerobic metabolism by inducing the activity of three tricarboxylic acid (TCA) cycle dehydrogenases [66]. Thus, MAMs effectively integrate Ca^2+^ flux with cellular metabolic pathways. In addition, MAMs also play a crucial role in mitochondrial morphology regulation. MAMs are rich in DRP1, and mitochondrial division is induced at sites of MAMs [67,68].

Aging leads to the disruption of Ca^2+^ homeostasis and increased ROS generation, and the Ca^2+^ and ROS signaling pathways overlap and interact with each other [69]. ROS can affect Ca^2+^ homeostasis. The mitochondrial Ca^2+^ uniporter (MCU) is the main Ca^2+^ channel, and it is regulated by ROS through S-glutathionylation of cysteine 97 (C97). Oxidation and mutation of C97 enhance the MCU channel activity, resulting in the elevation of the ROS and Ca^2+^ levels within mitochondria [70]. During ischemia/reperfusion, the redox-sensitive protein CaMKII is activated, increasing Ca^2+^ uptake via MCU and promoting myocardial death and mPTP opening [71]. Ca^2+^ can regulate the level of ROS. Studies have shown that defective ER-to-mitochondrial Ca^2+^ signaling increases mitochondrial Ca^2+^ levels, causing oxidative stress [72]. Ca^2+^ diminishes ROS leakage from complexes I and III under physiological conditions but increases ROS production during complex blockage [73]. Furthermore, mitochondrial Ca^2+^ uptake may cause a mild decrease in the mitochondrial membrane potential, which may lead to increased ROS production via alterations in the pH gradient at the inner mitochondrial membrane [74]. In conclusion, Ca^2+^ affects mitochondrial ROS production, while ROS modulate the activity of proteins to ensure and control Ca^2+^ flux between cellular compartments.

Decreased numbers of MAMs in senescent cells lead to a decrease in the ability of mitochondria and endoplasmic reticulum to coordinately regulate [75]. Aging-associated reduced ER content may lead to reduced MAMs, which can lead to decreased mitophagy and trigger mitochondrial dysfunction [75,76]. Alterations in the mitochondrial–ER calcium flux can also affect aging in mice. Deletion of the mouse ER calcium channel inositol 1,4,5-trisphosphate receptor (ITPR or IP3R) reduced age-related alterations in MAMs [77]. Ca^2+^ may be an initiator of mitochondrial failure, leading to synaptic defects and neurodegeneration observed during aging [78]. Furthermore, Cherubini et al. [79] demonstrated that abnormal DRP1-mediated mitochondrial fragmentation in the striatum of a mouse model of Huntington’s disease caused mitochondria to move away from the ER and disrupted the mitochondrial–ER binding, thus causing defects in Ca efflux and overproduction of mitochondrial superoxide species. The above evidence suggests that aging can lead to changes in MAMs, and targeted regulation of MAMs may be a target for potentially delaying aging to promote health.

Recently, exercise has been shown to promote health by regulating MAMs. Audrey Merle et al. [80] demonstrated that aerobic exercise induces a significant upregulation of developmental regulation and DNA damage 1 (REDD1) expression and that mitochondria–ER interactions are reduced. Another study confirmed that swimming training prolonged the lifespan of amyotrophic lateral sclerosis (ALS) mice accompanied by changes in MAM components, such as lower mitochondrial cholesterol and enhanced caveolin-1 expression [81]. Exercise also mediates skeletal muscle health benefits by modulating calcium signaling. Ca^2+^ regulation is one of the main functions of MAMs, and exercise may improve the regulation of Ca^2+^ homeostasis by altering MAMs. Boncompagni et al. [82] demonstrated that exercise prevented the inappropriate accumulation of stromal interaction molecule (STIM) 1 and ORAI1 in ER tubular aggregates during aging, and STIM1 and ORAI1 are the two main proteins that participated instore-operated Ca^2+^ entry (SOCE), maintaining the ability of aging muscles to replenish intracellular Ca^2+^ stores through SOCE. STIM1 is a positive regulator of ITPR3 gene expression, and ITPR3 is enriched in the MAMs involved in the regulation of Ca^2+^ homeostasis [83,84]. The above evidence suggests that exercise may mediate the health benefits of exercise by modulating MAMs and promote mitochondrial homeostasis and skeletal muscle health by regulating Ca^2+^ homeostasis through MAMs.

## 3. Mitochondria-Associated Epigenetic Changes during Skeletal Muscle Aging

A complex relationship exists between epigenetics and aging, and epigenetic modifications can affect all tissues and cells throughout the life cycle [85]. Changes in epigenetic modifications, such as DNA methylation and histone modifications, have become hallmarks of aging [8]. The dimorphism of the distribution of muscle fiber types in different sexes suggests that differences in the epigenetic changes of skeletal muscle potentially exist between males and females during aging [27].

### 3.1. Aging-Associated Alterations in DNA Methylation

Skeletal muscle undergoes DNA methylation changes across the lifespan [86,87], and DNA methylation as a marker of aging has been used as an epigenetic clock to predict the physiological age of organisms [88]. DNA methylation downregulates gene expression by inhibiting transcriptional processes. Exercise generally decreases the level of DNA methylation in skeletal muscle, upregulates gene expression, and promotes skeletal muscle adaptation [13]. Substantial evidence indicates that skeletal muscle aging can be delayed by targeting the mitochondria–proteostasis axis. Therefore, altered DNA methylation of nuclear genes encoding mitochondria-associated proteins and genes regulating mitochondrial function may contribute to the skeletal muscle aging phenotype by affecting mitochondrial quality or function. Sex differences in skeletal muscle phenotypes are noted during aging. Davegardh et al. [89] demonstrated differences in SIRT1 and KDM6A gene DNA methylation in myoblasts derived from different sexes, and the study suggested that differences in DNA methylation may lead to sex-dependent differences in skeletal muscle phenotypes. Koczor et al. [90] found that PGC-1α transcription was reduced by approximately 65% in aging skeletal muscle, accompanied by a decrease in PGC-1α mRNA stability, and altered expression patterns of PGC-1α transcriptional regulators, including nuclear factor erythroid 2-related factor 2 (NRF2), undifferentiated embryonic cell transcription factor 1 (UTF1), ATF2, and yin yang 1, in aging skeletal muscle. Additionally, increased nuclear DNA methylation levels were found in aging skeletal muscle, and DNA methyltransferase (DNMT) 3b protein levels were 1.9-fold higher than those in young skeletal muscle. Therefore, aging may affect mitochondrial biogenesis and skeletal muscle health by increasing DNMT3b expression, upregulating nuclear genome methylation levels, and downregulating PGC-1α protein expression. This idea can be supported by the study of the myocardium by Rawat et al. [91] who found that doxorubicin treatment affected DNMT protein levels, which downregulated the expression of transcription factors, such as PGC-1α, and thus affected mitochondrial function. In addition, UPRmt is regulated by DNA methylation. DNA methylation in the ATF5 promoter region was decreased, and ATF5 mRNA expression was elevated in glioma tissues compared with normal tissues [92]. Similarly, in HCC tissues, the ATF5 promoter region was hypermethylated, and ATF5 mRNA and protein expression levels were downregulated [93]. Mitochondrial homeostasis-related genes are regulated by DNA methylation. Aging leads to changes in DNA methylation, affecting mitochondrial homeostasis-related gene expression, which subsequently leads to mitochondrial dysfunction.

### 3.2. Aging-Associated Alterations in Histone Posttranslational Modifications

Benayoun et al. [10] summarized the changes in histone posttranslational modifications (hPTMs) found in different biological aging organisms, such as *C. elegans*, flies, mice, and *Homo sapiens*, and found that histone modifications H3K9me1, H4K20me2, H4K20me3, and H3K4me3 were upregulated in aging individuals. In contrast, H3K9me2, H3K9me3, H4K27me3, H4K56ac, and H4K16ac were downregulated in aging individuals. Similarly, targeted regulation of histone methylation-modifying enzymes regulates the lifespan through histone modifications, such as sirtuin (SIRT) 1, SIRT6, silent information regulator (SIR) 2, and general control nonderepressible (GCN) 5, upregulating the lifespan in different species. In contrast, UTF1, Jumonji domain-containing protein (JMJD) 2, lysine-specific demethylase 1, SET9, SET15, serum antigenic substance (SAS) 3, and SAS2 downregulate the lifespan [10]. The above evidence shows that aging is associated with a variety of apparent modifying enzymes and histone modifications. Nagarajan et al. [11] demonstrated that histone acetyltransferase 1 (HAT1) expression was decreased in the skeletal muscle of aged mice and found that HAT1 knockout mouse models showed characteristics of early onset of aging-associated phenotypes such as mitochondrial dysfunction and muscle atrophy. When considering the inextricable relationship between mitochondria and aging, it can be hypothesized that histone methylation modification of mitochondrial protein-related genes encoded by nuclear DNA is also related to skeletal muscle aging. In addition, UPRmt activation is regulated by histone modifications. Various histone-modifying enzymes regulate mitochondrial homeostasis and longevity by affecting UPRmt, such as histone methyltransferases microbial ecosystem therapeutic-2 (MET-2) and nuclear cofactor lin-65 [94], histone lysine demethylases JMJD-1.2/PHF8 and JMJD-3.1/JMJD3 [95], acetyltransferase CREB-binding protein 1 [96], and HDAC1 [97]. These findings suggest that epigenetic mechanisms may play an important role in the aging-induced suppression of UPRmt activation. Therefore, targeting histone modifications may be an effective method of improving mitochondrial homeostasis and promoting skeletal muscle health.

### 3.3. Aging-Associated Alterations in miRNA Expression

With increasing age, microRNAs (miRNAs) in organisms appear to be differentially expressed or have dysregulated activity, so miRNAs have the potential to be aging markers or aging modulators. As early as in 2008, Drummond et al. [98] demonstrated that pri-miR-1-1, pri-miR-1-2, pri-miR-133a-1, and pri-miR-133a-2 expression was elevated in older men compared to younger men. Jia et al. [99] similarly found that miR-133a, miR-133c, miR-192, and miR-151-3p may play an important role in muscle growth and development in sika deer skeletal muscle, and miR-17-5p, miR-378b, miR-199a-5p, and miR-7 may play a key role in skeletal muscle aging. The abundant miR-133a in skeletal muscle plays an important role in mitochondrial function and the aging process of skeletal muscle. Moreover, a sex difference in miR-133a and miR-133b content in the quiescence state was observed [100]. These results suggest that there may be sex differences in miRNA regulation. In addition, miR-131a can also regulate muscle development. The absence of miR-131a in mouse skeletal muscle induces a decrease in mitochondrial biogenesis, basal metabolic rate, and exercise capacity [101]. Thus, miR-131a can mediate the development of aging by affecting mitochondrial biogenesis and mitochondrial function. Mitochondrial dysfunction in aged skeletal muscle is also an important risk factor for sarcopenia [7]. Goljanek-Whysall et al. [4], established that miR-181a is an endogenous regulator of mitochondrial dynamics. The expression of miR-181a is downregulated during aging, leading to an increase in abnormal mitochondria and activating mitophagy-related proteins, whereas miR-181a overexpression prevents the accumulation of p62, DJ-1, and PARK2 and improve mitochondrial dynamics and skeletal muscle function. Several studies have confirmed that UPRmt is regulated by miRNAs. Dahlmans et al. [102] demonstrated that silencing miR-382 in C2C12 myotubes induces mitochondrial nucleoprotein imbalance and activates UPRmt. In heart failure cardiomyocytes after ischemia, miR-129-5p and miR-489 regulate UPRmt by targeting ATF5 and LONP1 [103].

**Figure 1:**
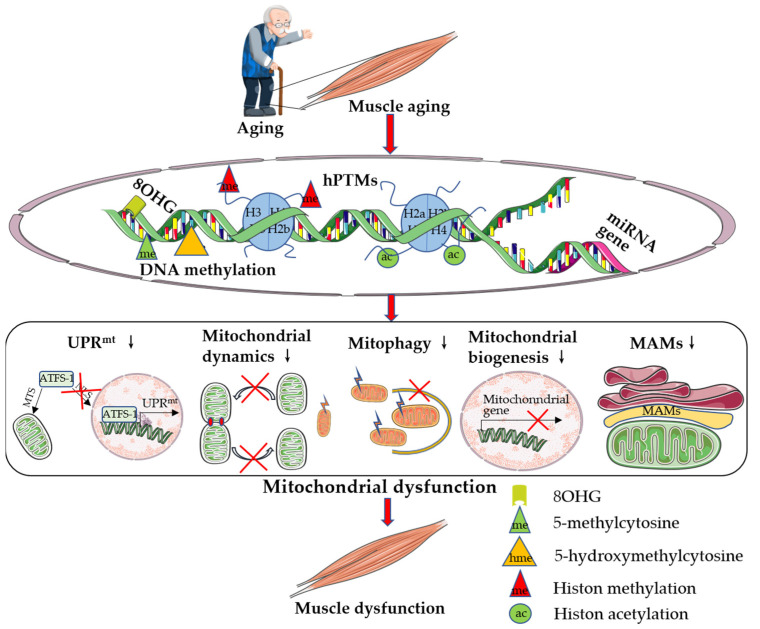
Aging induces skeletal muscle mitochondrial dysfunction via epigenetic modifications. Skeletal muscle aging affects nuclear genomic DNA methylation, hPTMs, and miRNA expression, which subsequently inhibit mitochondrial quality control, leading to mitochondrial and skeletal muscle dysfunction. For abbreviations, see the list at the end of the paper.

In conclusion, nuclear DNA methylation, histone modifications, and miRNA modifications in skeletal muscle are all altered with increasing age. Alterations in these epigenetic modifications subsequently induce skeletal muscle dysfunction by downregulating mitochondrial content and function (Figure 1). Research evidence shows that genes that target and regulate nuclear DNA encoding mitochondria-related proteins can induce skeletal muscle health and prevent skeletal muscle aging by upregulating mitochondrial content and function.

## 4. Exercise Mitigates Skeletal Muscle Aging via the Regulation of Mitochondria-Associated Epigenetics

### 4.1. Exercise-Induced Alterations in DNA Methylation

Accumulating evidence suggests that exercise-induced mitochondria-associated DNA methylation plays an important role in the health benefits of exercise. Research in the field of exercise training and DNA methylation has shown that the main regulator of mitochondrial biogenesis is PGC-1α. As early as in 2012, Barres et al. [104] demonstrated that promoter methylation of PGC-1α, TFAM, myocyte enhancer factor (MEF) 2A, and pyruvate dehydrogenase kinase 4 (PDK4) was significantly reduced in the soleus muscle after acute exercise and that the PPAR-δ gene showed delayed hypomethylation 3 h after exercise. Bajpeyi et al. [105] similarly demonstrated that after acute exercise in humans, demethylation of the skeletal muscle PGC-1α-260 CpG site, upregulation of the PGC-1α mRNA expression, and increased mitochondrial biogenesis are noted. Hunter et al. [106] demonstrated that DNMT3a and DNMT3b mRNA expression decreased after exercise and that the degree of genome-wide DNA methylation and methylation of the PGC-1α gene promoter was reduced. In addition, sex-related differences in exercise-induced DNA methylation changes in skeletal muscle potentially occur [107]. A meta-analysis identified that DNA methylation changes to a greater extent in females than in males after exercise, suggesting sex differences in epigenetic responses to exercise [108]. These studies all suggest that exercise can affect mitochondrial biogenesis by altering the DNA methylation of the genes involved in mitochondrial biogenesis in skeletal muscle. Maasar et al. [109] found that young male athletes had reduced methylation of genes related to adenosine 5′-monophosphate-activated protein kinase (AMPK), mitogen-activated protein kinase, protein binding, insulin, and axonal guidance pathways after 30 min of exercise and increased PGC-1α expression. In addition, Rasmussen et al. [110] demonstrated that 20 min after a single exercise, methylation of the promoter of the TFAM gene, a regulator of mtDNA transcription and replication, decreased, and TFAM mRNA levels increased. In addition, Small et al. [111] demonstrated that the DNA methylation of genes related to muscle development decreased in skeletal muscle in DNMT3A knockout mice. Therefore, we hypothesize that exercise may upregulate mitochondrial biogenesis by downregulating the expression of DNMTs and reducing the methylation levels of mitochondria-related genes, such as PGC-1α and TFAM, resulting in skeletal muscle health benefits. Based on the above evidence, exercise may regulate mitochondrial content, quality, and function by inducing changes in mitochondria-related DNA methylation modification patterns in skeletal muscle, which subsequently promotes skeletal muscle health and prevents skeletal muscle aging.

### 4.2. Exercise-Induced Alterations in Histone Posttranslational Modifications

Exercise can affect gene expression and can regulate mitochondrial function by regulating epigenetic modifications such as histone methylation, acetylation, and ubiquitination. Exercise-induced PGC-1α gene expression is regulated not only by DNA methylation, but also by histone methylation. Lochmann et al. [112] demonstrated that in a mouse acute exercise model, the PGC-1α promoter transcriptional activity marker H3K4me3 levels increased 2–4-fold and the PGC-1α mRNA levels increased in the quadriceps femoris muscle. Exercise may improve skeletal muscle health by increasing H3K4me3 modification in the PGC-1α promoter, upregulating PGC-1α protein expression, and increasing mitochondrial biogenesis. Furthermore, exercise can also regulate gene expression by altering histone acetylation modifications. Exercise-induced SIRT1 expression, which regulates PGC-1α expression through epigenetic modifications, has been widely reported. Exercise-induced PGC-1α expression may be regulated by histone deacetylases (HDACs) [113]. Exercise increased the expression of the NRF1 and MEF2A genes by inducing increased acetylation in the NRF1 and MEF2A promoters [114], and two MEF2 binding sites on the PGC-1α gene promoter were noted. MEF2 is an upstream regulator of PGC-1α and upregulates the expression of PGC-1α [115]. In addition, Masuzawa et al. [116] found that 2 h after acute exercise in rats, the level of histone 3 acetylation at the proximal promoter of PGC-1α increased in a muscle fiber type-dependent manner, which was accompanied by an increase in the level of PGC-1α mRNA. Taken together, these studies linking exercise to posttranslational modifications of mitochondria-related genes histones suggest that exercise-induced regulation of posttranslational modifications of mitochondria-related genomic proteins may be an important link in the exercise-induced adaptive responses in skeletal muscle. Exercise may promote skeletal muscle health and prevent skeletal muscle aging by altering hPTMs.

### 4.3. Exercise-Induced Alterations in miRNA Expression

In addition to DNA methylation and hPTMs, miRNA-mediated epigenetic regulation may alter gene expression and affect protein translation processes through posttranscriptional regulation. Those miRNAs that derive from skeletal or cardiac muscle are termed muscle-specific microRNAs (myomiRs). Only seven myomiRs associated with skeletal muscle have been identified, namely, miR-1, miR-133a, miR-133b, miR-206 (expressed only in skeletal muscle), miR-208b, miR-486, and miR-499, and the expression levels of these miRNAs depend on exercise intensity [117]. Russell et al. [118] demonstrated that exercise not only upregulates the expression level of myomiRs, but can also increase the expression of miRNA maturation-related proteins such as Drosha, Dicer, and exportin-5. Rodrigues et al. [119] demonstrated by skeletal muscle injection of miR-1 that miR-1 overexpression can activate p-AMPK, upregulate PGC-1α and carnitine palmitoyltransferase 1b protein expression, and improve skeletal muscle mitochondrial biogenesis and skeletal muscle oxidative metabolism. Nie et al. [101] demonstrated that 6 weeks of endurance exercise increased the level of miR-133a and increased the expression of the mitochondrial biogenesis regulators PGC-1α, peroxisome proliferative activated receptor gamma coactivator-1β (PGC-1β), NRF1, and TFAM in skeletal muscle of wild-type mice, followed by upregulation of mitochondrial biogenesis. In addition, the researchers showed that exercise-induced mitochondrial biogenesis was blocked in miR-133a knockout mice. These results suggest that miR-133 plays an important role in regulating exercise-induced skeletal muscle adaptation and health. In addition to myomiRs, exercise may also regulate mitochondria by regulating the expression of other types of miRNAs. Sun et al. [120] demonstrated that miR-494 and miR-696 were significantly decreased in the gastrocnemius muscle, accompanied by increased NRF1 mRNA and PGC-1α protein expression after 8 weeks of spontaneous wheel running exercise in mice, suggesting that exercise may regulate mitochondrial biogenesis by regulating miRNA expression. Recently, Massart et al. [121] demonstrated that miR-19b-3p expression was increased in the skeletal muscle of both humans and mice after endurance exercise. Overexpression of miR-19b-3p in mouse skeletal muscle can upregulate AMPKα and mitochondrial complex subunits and increase glucose transport and OXPHOS [121]. To date, there has been no research on miRNA differences in skeletal muscle of different sexes after exercise, but one study found sex differences in miR-4675, miR-6745, and miR-6746-3p in saliva after long-distance running [122].

In conclusion, exercise can regulate mitochondrial content and function through epigenetic modifications such as DNA methylation, hPTMs, and miRNA expression, which subsequently maintain the health of skeletal muscle and prevent skeletal muscle aging. Therefore, different types of epigenetic modifications represent potential targets for preventing skeletal muscle aging and treating skeletal muscle-related diseases.

## 5. Role of ROS in the Epigenetic Modification of Skeletal Muscle Mitochondria

**Figure 2:**
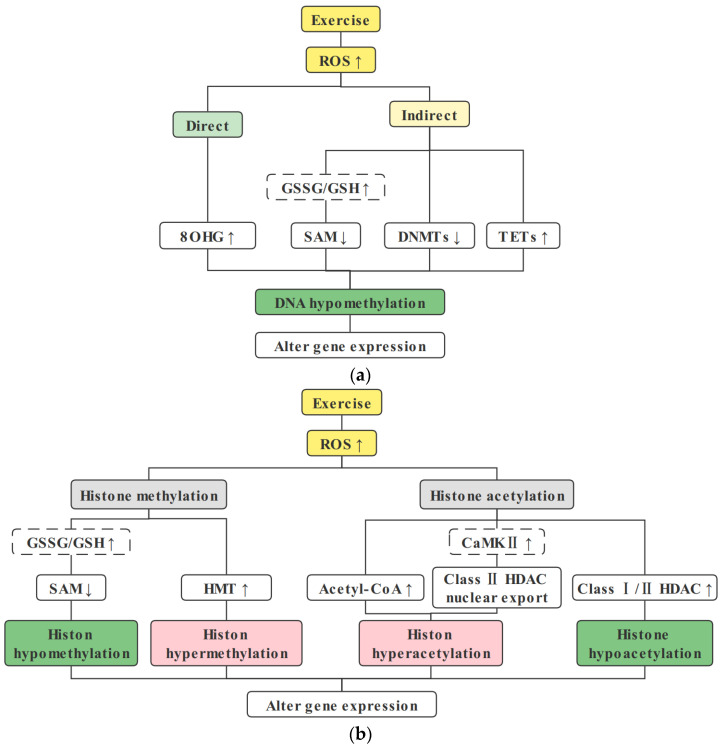

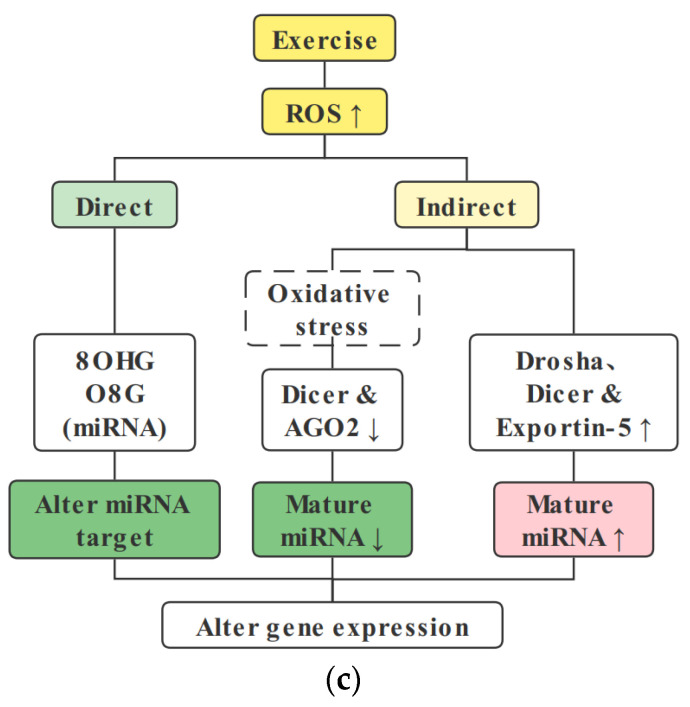
Exercise-induced ROS regulate epigenetic modifications. (**a**) Exercise-induced ROS downregulate DNA methylation. ROS can directly inhibit DNA methylation via the formation of 8OHG; ROS can inhibit DNA methylation by downregulating the availability of SAM; ROS can also regulate the activity and/or expression of DNMTs and TETs, inducing a decrease in DNA methylation. (**b**) Exercise-induced ROS can affect hPTMs. ROS can decrease histone methylation by downregulating SAM availability; ROS can increase histone methylation modification by upregulating HMTs; ROS can increase histone acetylation by upregulating acetyl-CoA availability or by inducing class II HDAC nuclear export through CaMKII; and ROS downregulate histone acetylation by upregulating class I/II HDACs. (**c**) Exercise-induced ROS regulate miRNA expression. ROS can directly oxidize miRNA and change the target mRNAs of miRNA; ROS can promote miRNA biogenesis by upregulating Drosha, Dicer, and extrotin-5; however, acute high-intensity exercise-induced ROS may cause oxidative stress and inhibit miRNA biogenesis by downregulating Dicer and AGO2. For abbreviations, see the list at the end of the paper.

Skeletal muscle is the major energy-consuming organ, and the required ATP is mainly provided by mitochondrial aerobic metabolism. During mitochondrial aerobic respiration to generate ATP, ROS are inevitably generated as byproducts. ROS are mainly produced by mitochondria. ROS in cells play a bidirectional regulatory role, and physiological levels of ROS play a crucial role in exercise-mediated signal transduction in skeletal muscle cells. However, ROS beyond the antioxidant capacity of cells can oxidize proteins, lipids, DNA, and RNA, causing oxidative stress. ROS act as regulators of epigenetic modifications and regulate gene expression by regulating epigenetic modifications such as DNA methylation, hPTMs, and miRNA expression [15,123,124]. Recent studies have shown that exercise-induced physiological levels of ROS lead to extensive changes in the epigenome of skeletal muscle cells, modulating gene expression and promoting skeletal muscle health benefits. Exercise appears to promote mitochondrial health by inducing ROS to alter mitochondrial-related gene epigenetic modifications (Figure 2).

### 5.1. ROS Are Necessary for Exercise-Induced Skeletal Muscle Health Benefits

In the classic view, ROS act as a double-edged sword in cellular processes; excess ROS can cause oxidative stress, and physiological levels of ROS play an essential role in cell signaling [125,126,127]. Skeletal muscle ROS induced by regular exercise play an essential role in cellular signaling pathways that promote adaptive changes in skeletal muscle, such as increasing protein synthesis, activating insulin signaling, inducing mitochondrial biogenesis, activating signaling pathways, controlling skeletal muscle production, affecting gene expression, and positively regulating antioxidants [128]. Although previous studies have suggested that antioxidant supplementation is beneficial for health, the current mainstream view is that antioxidant supplementation during exercise does not promote adaptive skeletal muscle changes but can even inhibit exercise-mediated health benefits. For example, Ristow et al. [129] demonstrated that exercise-induced ROS may play a role in promoting insulin sensitivity and antioxidant defense in humans by upregulating PGC-1α, PGC-1β, SOD1, SOD2, and glutathione peroxidase 1. Daily intake of vitamins C and E almost completely eliminated the changes in the expression of these genes and the increase in insulin sensitivity after exercise [129]. Recently, Wang et al. [130] demonstrated that regular exercise could activate UPRmt and increase the content of SOD2. However, the expression of c-JUN, the C/EBP homologous protein, HSP60, CLpP, SIRT3, high-temperature-regulated A2 (also known as OMI), and SOD2 decreased after intervention with mitochondrial-derived ROS (mtROS) with the mitochondria-targeted antioxidant MitoTEMPO. These results suggest that mtROS are a key factor for exercise to promote UPRmt. Thus, antioxidant supplements prevent many of the beneficial effects of exercise on metabolism. This evidence demonstrates that ROS are necessary for the exercise-induced health effects in skeletal muscle. We cannot deny that oxidative stress caused by aging induces changes in epigenetic modifications in skeletal muscle [15], whereas exercise can reduce aging-related oxidative stress by upregulating the antioxidant capacity of skeletal muscle [131,132]. Considering that exercise-induced ROS are a necessary condition for exercise-induced skeletal muscle health, we paid more attention to the biological role of exercise-induced ROS as signaling molecules in the regulation of skeletal muscle epigenetic modifications.

### 5.2. ROS Regulate DNA Methylation

ROS can alter DNA methylation patterns through multiple pathways. High levels of ROS cause oxidative DNA damage, and then DNMT1 and poly ADP-ribose polymerase 1 are recruited to specific recognition sites to induce gene promoter methylation. However prolonged ROS exposure induces demethylation by catalyzing the conversion of 5-methylcytosine (5-mC) to 5-hydroxymethylcytosine (5-hmC) by upregulating ten–eleven translocation (TET) enzyme s [133]. ROS can also oxidize guanosine to 8-oxo-7,8-dihydro-2′-deoxyguanosine (8OHG) to affect DNA methylation. When 8OHG persists, it inhibits DNA methylation, and 8-oxoguanine DNA glycosylase (OGG) 1 attached to 8OHG promotes DNA demethylation by recruiting TET1, resulting in DNA hypomethylation and transcriptional activation [14,134,135]. Exposure to ROS upregulates cellular TET activity and promotes the conversion of 5-mc to 5-hmc, so TETs can be used as a link between cellular redox status and epigenome maintenance [136]. ROS can also modulate S-adenosylmethionine (SAM) availability, affecting DNA methylation and histone methylation modifications. ROS decrease SAM synthesis by inhibiting methionine synthase and methionine adenosyltransferase; on the other hand, the methionine residue generates glutathione (GSH) through cysteine metabolism, which further reduces SAM synthesis and downregulates DNA methylation and histone methylation [15,137] (Figure 2a).

Strenuous exercise induces increased ROS production [138], leading to GSH depletion, which alters the glutathione disulfide (GSSG)/GSH ratio, affects SAM availability, and downregulates DNA methylation modifications. Stephens et al. [139] demonstrated that 10 weeks of aerobic exercise can downregulate the methylation of promoters of genes related to mitochondrial function and glutathione metabolism. The above studies showed that exercise-induced ROS may affect mitochondrial epigenetic modifications and promote mitochondrial function by altering the GSSG/GSH ratio. Hunter et al. [106] demonstrated that DNMT3a and DNMT3b mRNA expression was decreased after exercise and that both genome-wide DNA methylation and methylation of the PGC-1α gene promoter were decreased. Considering that ROS regulate DNMT protein expression, exercise-induced ROS may improve mitochondrial function by downregulating the expression of DNMTs and the DNA methylation of mitochondria-associated genes. Of course, exercise-induced ROS may also downregulate mitochondria-associated gene DNA methylation by increasing TET protein activity. Carter et al. [9], however, found that PGC-1α transcription was reduced by approximately 65% in aging skeletal muscle and was accompanied by reduced PGC-1α mRNA stability. Altered expression patterns of PGC-1α transcriptional regulators were also observed in aging muscle, including NRF 2, UTF 1, ATF 2, and yin yang 1. In addition, nuclear genome methylation levels were increased in aging muscle, with DNMT3b protein levels increased 1.9-fold compared with young muscle. Therefore, exercise-induced physiological levels of ROS may lead to hypomethylation of nuclear mitochondria-related genes via downregulation of DNMTs and upregulation of TETs, which promote mitochondrial function and skeletal muscle health, while aging-induced high levels of ROS may play the opposite role.

### 5.3. ROS Regulate Histone Posttranslational Modifications

ROS affect hPTMs by modulating epigenetic regulators and enzymatic activity. Class I/II HDAC activity is elevated under short-term oxidative stress conditions, resulting in reduced global histone acetylation [123]. Recently, Pradhan et al. [124] treated cells with sublethal doses of hydrogen peroxide (H_2_O_2_) and found enhanced expression of DNMT and HDAC, elevated DNA methylation of the cdc20 homolog 1 promoter, increased enrichment of H3K9me3 and H3K27me3 modifications, and inhibition of gene expression. ROS can also modulate histone acetylation modifications by modulating the availability of acetyl-coenzyme A (acetyl-CoA), a substrate for histone acetylation. Seo et al. [140] found that H_2_O_2_ treatment of cells increased glucose uptake and promoted increased acetyl-CoA levels. Marmisolle et al. [141] found that H_2_O_2_ reduces the mRNA level of acetyl-CoA carboxylase (ACC), an enzyme that catalyzes the ATP-dependent carboxylation of acetyl-CoA to malonyl-CoA and inhibits the consumption of acetyl-CoA. Exercise induces phosphorylation activation of calmodulin-dependent protein kinase II (CaMKII) and AMPK via ROS [142,143], induces phosphorylation-dependent nuclear export of class IIa HDACs, and increases histone acetylation modifications [144]. A slight increase in ROS concentration induces upregulation of the SIRT1 protein, whereas high levels of H_2_O_2_ result in SUMOylation and proteasomal degradation of the SIRT1 protein [145,146] (Figure 2b).

Furthermore, the acetylation level of H3 at the promoter of PGC-1α increased in a muscle fiber type-dependent manner at 2 h after acute exercise in rats, which was accompanied by an increase in PGC-1α mRNA levels [116]. ROS-induced increases in SIRT1 protein expression can also affect p53 and FOXO3a, increasing mitochondrial antioxidant capacity by upregulating SOD2 and catalase [145].

Histone methylation is similarly regulated by exercise and ROS. Histone methyltransferases (HMTs), such as DNMT, use SAM as a substrate to regulate histone methylation modifications, and SAM availability is regulated by ROS [15,137]. ROS can regulate multiple types of histone methylation modifications, including transcriptional activation markers H3K4me2/3 and transcriptional repression markers H3K9me2/3 and H3K27me3 [14]. H3K4 methylation is highly enriched at promoter regions and transcription start sites and increases with exercise [147]. Lochmann et al. [112] demonstrated that acute exercise in mice resulted in a 2–4-fold increase in the level of the transcriptional activity marker H3K4me3 at the PGC-1α promoter in the quadriceps femoris and an increase in the level of PGC-1α mRNA expression. The above evidence suggests that exercise-induced ROS can promote PGC-1α protein expression, increase mitochondrial biogenesis, and improve skeletal muscle health by upregulating the transcriptional activation marker H3K4me3 and histone acetylation.

### 5.4. ROS Regulate miRNA Expression

In addition to DNA methylation and histone modifications, miRNAs can also alter gene expression through posttranscriptional regulation and protein translation process regulation. ROS can also directly oxidize certain miRNAs and hydroxylate guanine to generate 8-oxo-7,8-dihydroguanosine (8OHG), altering its structure and stability [148]. Recent studies have found that miRNA-specific position 8-oxoguanine (O8G) modification can alter the target mRNA of miRNA and play an epigenetic regulatory role [149,150]. ROS can affect miRNA maturation by downregulating the expression of Dicer and posttranslationally modifying argonaute-2 (AGO2) and altering Dicer and AGO2 activities [151]. ROS can also alter miRNA expression by regulating DNMT1 and HDAC2 [152,153] (Figure 2c).

As early as in 2009, Safdar et al. [154] demonstrated that exercise upregulates the PGC-1α protein and promotes mitochondrial biogenesis by inhibiting the expression of miR-23, a negative regulator of PGC-1α mRNA. Subsequently, multiple studies have demonstrated that exercise can regulate the biogenesis and function of mitochondria by regulating various miRNAs, such as miR-133a, miR-494, miR-696, and miR-196-3p [101,120,121]. Exercise not only regulates the expression level of miRNA, but also increases the expression of miRNA biogenesis-related proteins such as Drosha, Dicer, and exportin-5 [118]. ROS can regulate all processes of miRNA biogenesis [151]. Thus, exercise may regulate the expression or activity of miRNA biogenesis-related proteins, such as Dicer and AGO2, by inducing ROS, altering miRNA expression, promoting mitochondria-associated protein expression, and inducing skeletal muscle health.

In conclusion, ROS can affect multiple epigenetic modifications through different regulatory mechanisms according to concentration and exposure time, and the regulatory effect of ROS on the same epigenetic modification cannot be generalized. The physiological level of ROS induced by exercise can alter mitochondria-related epigenetic modifications, upregulate mitochondrial protein expression, and promote mitochondrial health by affecting various epigenetic modifications, such as DNA methylation, hPTMs, and miRNA expression.

## 6. Exercise Modulates Myokine Expression

### 6.1. Exercise Reverses Myokine Expression during Aging

Skeletal muscle is the largest organ in the human body and is a highly differentiated tissue with high plasticity. Skeletal muscle is also a secretory organ. Cytokines and peptides whose production, expression, and release from muscle fibers are promoted by muscle contraction are called “myokines” [155]. According to Skel et al., muscle plays autocrine, paracrine, and endocrine roles and communicates with other organs, such as adipose tissue, liver, pancreas, bone, and brain, through myokines [156,157]. Scientists collectively refer to the exercise-induced signaling molecules produced by skeletal muscle, cardiac muscle, liver, and adipose tissue as “exerkines” [158]. Many myokines induced by muscle contraction belong to muscle-derived exerkines [159,160]. The contents of various myokines, such as interleukin (IL)-6, irisin, myostatin (MSTN), brain-derived neurotrophic factor (BDNF), and apelin, also change correspondingly with increasing age (Figure 1).

IL-6 was the first discovered and most extensively studied muscle factor [161]. Studies have shown that IL-6 is a double-edged sword with bidirectional regulatory effects of anti-inflammatory and proinflammatory effects [162,163]. Aging results in increased cellular IL-6 expression, and increased IL-1β and tumor necrosis factor TNF(TNF)-α expression exerts extensive proinflammatory effects, resulting in skeletal muscle atrophy [164]. However, IL-6 levels in skeletal muscle and plasma were also elevated after exercise [165]. Exercise is considered an anti-inflammatory intervention that can reduce or even reverse the muscle wasting process in cancer cachexia [164]. In tumor animal models, resistance training can increase the IL-10/TNF-α ratio and plasma IL-10 levels, exerting an anti-inflammatory effect [166]. Studies of marathoners have found that exercise leads to a significant increase in both IL-6 and IL-10 levels in plasma, exerting anti-inflammatory effects [167]. Exercise-induced IL-6 acts as an anti-inflammatory myokine by inhibiting TNF-α and improving glucose uptake by stimulating AMPK signaling [164]. Therefore, exercise-induced IL-6-mediated anti-inflammatory effects play an important role in exercise-induced skeletal muscle health. Li et al. found that mitochondrial respiration and enzyme activity were reduced in skeletal muscle and partially restored to normal after exercise training in an IL-6-deficient mouse model [168]. These results suggest that exercise-induced IL-6 has a regulatory effect on skeletal muscle mitochondria.

Irisin, discovered by the Spiegelman team [169], has been the most studied myokine in recent years. Irisin is a fragment that is produced when splicing or cutting fibronectin type III domain-containing 5 (FNDC5), which has the function of converting white to brown fat and increasing skeletal muscle glucose uptake. Studies have shown that circulating irisin levels are associated with obesity, glucose tolerance, and insulin resistance status in the middle-aged Chinese population [170]. Irisin expression correlates with age and exercise. With increasing age and the occurrence of muscle atrophy in aged mice and elderly humans, the level of circulating irisin decreases, and both endurance exercise and resistance exercise can upregulate irisin expression and the circulating irisin content in the skeletal muscle of aging individuals [171,172,173,174,175]. Resistance exercise induced a greater irisin response than endurance exercise [176]. Exercise upregulates FNDC5/irisin expression via PGC1a, enhances mitochondrial fission and mitophagy, and improves myopathy following critical limb ischemia in aged mice [177].

MSTN plays an important role in skeletal muscle atrophy by increasing protein degradation [178]. MSTN increases with age, leading to the degeneration of skeletal and smooth muscle [179]. Exercise can reduce the levels of skeletal muscle and circulating MSTN [180,181,182], prevent T2DM and muscle atrophy, and protect skeletal muscle. Recent studies have shown that MSTN may induce an abnormal increase in mitochondrial fission by upregulating DRP1 protein expression, leading to skeletal muscle dysfunction in chronic obstructive pulmonary disease [183]. Therefore, exercise may improve mitochondrial homeostasis and promote skeletal muscle health by downregulating MSTN expression.

BDNF levels also decreased with age [184]. Decreased BDNF is associated with age-related hippocampal dysfunction, memory impairment, and an increased risk of depression, and exercise prevents aging-induced cognitive dysfunction by activating the hippocampal PGC-1α/FNDC5/BDNF pathway [185]. In addition, exercise induces an increase in skeletal muscle and circulating BDNF expression, which plays a role in the regulation of mitochondrial function in muscle and other tissues by activating AMPK [186,187].

Recently, apelin has been shown to play an important role in combating age-related muscle atrophy, activating AMPK, promoting mitochondrial biogenesis, and stimulating skeletal muscle regeneration [188]. Apelin decreases with age, and skeletal muscle contraction leads to increased apelin production [188,189]. In addition, maternal exercise during pregnancy increases skeletal muscle function in offspring mice by increasing apelin signaling and mitochondrial biogenesis [190].

In conclusion, a variety of myokines change with age, and exercise, as a nonpharmacological treatment, can effectively reverse age-related changes in myokines, regulate mitochondrial function, promote skeletal muscle health, and prolong lifespan.

### 6.2. Exercise Modulates Myokine Expression via Epigenetic Regulation

Myokine expression changes with increasing age. Exercise can reverse the changes in myokines with age. However, the mechanism by which exercise regulates myokine expression is not entirely clear. Exercise may directly regulate myokine gene DNA methylation, hPTMs, and miRNA expression and can regulate gene expression.

Exercise may upregulate IL-6 expression by altering epigenetic modifications. Exercise induced an increase in plasma IL-6 levels that was accompanied by a decrease in DNMT3b nuclear concentration in peripheral blood mononuclear cells, and a strong correlation was noted between a decrease in DNMT3b nuclear concentration and an increase in plasma IL-6 concentration after exercise [191]. It is suggested that exercise may increase the expression of IL-6 by reducing the nuclear concentration of DNMT3b and reducing DNA methylation. The study also showed that the nuclear concentration of DNMT3B decreased immediately after exercise by approximately 78% in young men, 72% in young women, 61% in adult men, and 53% in adult women [191]. Similarly, immediately after exercise, plasma IL-6 concentrations were increased approximately 35-fold in young men, 27-fold in young women, 25-fold in adult men, and 12-fold in adult women [191]. These results suggest that exercise-induced elevation of IL-6 and decreased nuclear concentration of DNMT3b are related to sex. Supplementation with different nutrients had different effects on the DNA methylation of the IL-6 gene with omega-3 polyunsaturated fatty acids increasing DNA methylation, whereas supplementation with extra virgin olive oil decreased DNA methylation [106]. In addition, IL-6 expression is also regulated by histone acetylation. Klymenko et al. [192] demonstrated that HDAC5 is a negative epigenetic regulator of IL-6 synthesis and release in skeletal muscle and that HDAC5 inhibits IL-6 expression by downregulating the IL6 promoter H3K9Ac modification. IL-6 expression is also regulated by miRNAs. The highly expressed miR-1, miR-146a, miR-181, and miR-155 in plasma were positively correlated with IL-6 levels after exercise [193]. IL-6 is a direct target of miR-223-3p, which can directly bind the 3′ untranslated region (UTR) of IL-6 and inhibit IL-6 expression [194,195]. The above evidence suggests that IL-6 expression is regulated by multiple epigenetic modifications.

Kim et al. [16] treated Huh7 cells with HDAC inhibitor sodium butyrate or DNA-demethylating agent 5-azacytidine and found increased H3 acetylation and decreased H3K27 methylation of the FNDC5 gene promoter and increased FNDC5 mRNA levels. These researchers showed that FNDC5 expression may be regulated by gene promoter DNA methylation and histone modifications. In addition, the expression of FNDC5/irisin is also regulated by ncRNAs. MiR-135a-5P inhibits the expression of FNDC5 [196]. Acting as a sponge of miR-135a-5p, circRNA ATF4 could upregulate the expression of FNDC5/irisin [17]. Exercise induced neurogenesis in the mouse hippocampus and the proliferation of neural precursor cells of the dentate gyrus of aged mice by downregulating miR-135a-5p [197]. Therefore, exercise may promote FNDC5/irisin expression by regulating epigenetic modification of the FNDC5/irisin gene.

Exercise can reduce the levels of skeletal muscle and circulating MSTN [180,181,182]. Fan et al. [198] demonstrated that sulforaphane treatment of porcine satellite cells inhibited MSTN expression by reducing histone acetylation enrichment in myogenic determination gene number 1 binding sites in the MSTN promoter. Low-protein diets fed to sows during gestation and lactation led to significant enrichment of MSTN gene promoters H3K9Ac and H3K4me3, upregulation of MSTN expression, and slow skeletal muscle growth of weaning piglets [18]. Treatment of C2C12 cells with siRNA targeting the MSTN gene promoter yielded enrichment of H3K9me2 in the MSTN promoter, resulting in silencing of MSTN [199]. MSTN expression is also regulated by miRNAs. Inhibition of miR-143 resulted in a 50% reduction in MSTN expression [200]. MiR-499 and miR-208b may synergistically target MSTN transcripts and downregulate MSTN expression [201]. Therefore, exercise may promote MSTN expression by regulating hPTMs and miRNA expression.

A single exercise bout reduces DNMT1 and DNMT3b levels in the adult rat hippocampus, downregulates DNA methylation in BDNF gene promoter IV, and increases BDNF gene expression [19]. Similarly, short-term exercise could alter the DNA methylation pattern of the BDNF gene and increase BDNF mRNA levels in the mouse hippocampus [202]. BDNF expression is also regulated by histone modifications. Exercise can induce an increase in the acetylation level of H3 and H4K8 in the BDNF gene promoter and upregulate BDNF expression [203,204]. Exercise can also inhibit HDAC2/HDAC3 through β-hydroxybutyrate followed by increased histone H3 acetylation to upregulate BDNF expression [205]. In addition, BDNF expression is also regulated by miRNAs [20]. For example, miR-140, miR-211, and miR-103a in human astrocytes [206,207,208] and miR-206 in the mouse brain [207] can regulate BDNF expression. Exercise also upregulated BDNF expression by inducing miRNAs. Running wheel exercise can recover hippocampal-related cognitive deficits caused by traumatic brain injury in mice, and the recovery process is related to miR-21 and miR-34a [209]. Therefore, exercise could promote BDNF expression by regulating the epigenetic modification of the BDNF gene.

Animal studies have shown that apelin expression is regulated by DNA methylation. Using high-throughput sequencing, Mishra et al. [210] found that greater than 10% DNA methylation of the CpG island of the apelin gene, resulting in a 5.9-fold reduction in apelin expression. Rat ozone exposure decreased the DNMT activity, and Dnmt3a/b gene expression increased DNA methylation in the apelin gene promoter and decreased apelin expression [211]. Contrasting findings have also been reported. Keleher et al. [212] demonstrated that hepatic apelin gene promoter DNA methylation was increased in the daughters of high-fat-fed mothers, but apelin was highly expressed. In addition, miR-224, miR-765, and miR-195 inhibit apelin expression [213,214,215]. Recent studies have demonstrated that miR-122-5P negatively regulates autophagy, apoptosis, and oxidative stress in rat cardiac fibroblasts by regulating the apelin–AMPK–mTOR signaling pathway [216], and miR-122-5p can also promote tumorigenesis and cardiac remodeling in hypertensive rats by regulating the ELABELA/apelin–apelin receptor axis and angiotensin-converting enzyme 2 (ACE2)–growth differentiation factor 15 (GDF15)–porimin signaling pathways [21].

In conclusion, the expression of various myokines is directly or indirectly regulated by epigenetic modifications. There is reason to believe that exercise may directly or indirectly upregulate the expression of myokines through different epigenetic modification patterns (DNA methylation, histone modifications, and miRNA) in promoting health. Exercise-induced ROS play an important role in the epigenetic regulation mentioned above, and may alter the expression of myokines by regulating the epigenetic modification of myokine genes.

## 7. Conclusions

**Figure 3:**
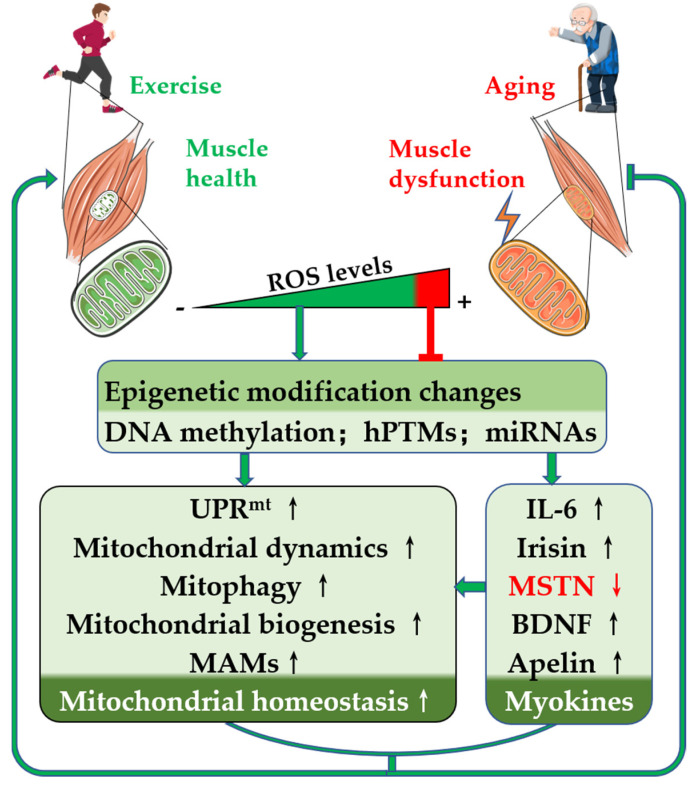
Exercise-induced ROS ameliorate aging skeletal muscle dysfunction via epigenetic regulation of mitochondrial homeostasis. With increasing age and decreasing exercise, ROS accumulation in skeletal muscle occurs. High ROS levels promote mitochondrial and skeletal muscle dysfunction. Exercise-induced physiological levels of ROS promote mitochondrial quality control and improve myokine expression by altering epigenetic modifications, which subsequently maintain mitochondrial homeostasis and promote skeletal muscle health. For abbreviations, see the list at the end of the paper.

As an epigenetic modulator, ROS can regulate a variety of epigenetic modification markers by oxidizing DNA and RNA directly or by affecting the expression and/or activity of a variety of epigenetic modification enzymes, thereby regulating mitochondrial quality and function. ROS are central signaling molecules that regulate cell survival and adaptation, and both aging and exercise can cause changes in cellular ROS. Aging, as a degenerative process, causes a prolonged and sustained increase in ROS and induces mitochondrial dysfunction in muscle. Exercise increases ROS production in contracting muscle, which acts as a signaling molecule to regulate muscle mitochondria-associated epigenetic markers and promote mitochondrial quality control. Mitochondrial biogenesis, mitochondrial dynamics, and mitophagy are all epigenetically regulated. In addition, as an endocrine organ, exercise can stimulate skeletal muscle to produce different types of myokines, mediate communication between muscle and other organs, and promote skeletal muscle health. Myokine expression is regulated by DNA methylation, hPTMs, and miRNA expression. The degenerative process of aging affects the expression of myokines, but exercise may reverse the changes in myokine expression by inducing ROS-mediated changes in epigenetic markers, thereby promoting muscle health and preventing or delaying the harmful effects of muscle aging. In conclusion, exercise, as a nondrug intervention, can induce changes in epigenetic markers via ROS, regulate the expression of mitochondrial homeostasis-related proteins, and maintain or enhance skeletal muscle mitochondrial health (Figure 3).

## Data Availability

Not applicable.

## Abbreviations

Acetyl-CoA	Acetyl-coenzyme A
AGO2	Argonaute-2
BDNF	Brain-derived neurotrophic factor
CaMKII	Calmodulin-dependent protein kinase II
DNMTs	DNA methyltransferases
GSH	Glutathione
GSSG	Glutathione disulfide
HDAC	Histone deacetylase
HMT	Histone methyltransferase
hPTMs	Histone posttranslational modifications
IL-6	Interleukin-6
miRNA	MicroRNA
MAMs	Mitochondria-associated endoplasmic reticulum membranes
MSTN	Myostatin
O8G	8-oxoguanine
8OHG	8-oxo-7,8-dihydroguanosine
ROS	Reactive oxygen species
SAM	S-adenosylmethionine
TETs	Ten–eleven translocation enzymes
UPR^mt^	Mitochondrial unfolded protein response

## References

[1] Kujoth G.C., Hiona A., Pugh T.D., Someya S., Panzer K., Wohlgemuth S.E., Hofer T., Seo A.Y., Sullivan R., Jobling W.A. (2005). Mitochondrial DNA Mutations, Oxidative Stress, and Apoptosis in Mammalian Aging. Science.

[2] Distefano G., Goodpaster B.H. (2018). Effects of Exercise and Aging on Skeletal Muscle. Cold Spring Harb. Perspect. Med..

[3] No M.-H., Heo J.-W., Yoo S.-Z., Kim C.-J., Park D.-H., Kang J.-H., Seo D.-Y., Han J., Kwak H.-B. (2020). Effects of aging and exercise training on mitochondrial function and apoptosis in the rat heart. Pflugers Arch..

[4] Goljanek-Whysall K., Soriano-Arroquia A., McCormick R., Chinda C., McDonagh B. (2020). miR-181a regulates p62/SQSTM1, parkin, and protein DJ-1 promoting mitochondrial dynamics in skeletal muscle aging. Aging Cell.

[5] Hood D.A., Memme J.M., Oliveira A.N., Triolo M. (2019). Maintenance of Skeletal Muscle Mitochondria in Health, Exercise, and Aging. Annu. Rev. Physiol..

[6] Cartee G.D., Hepple R.T., Bamman M.M., Zierath J.R. (2016). Exercise Promotes Healthy Aging of Skeletal Muscle. Cell Metab..

[7] Liu Q., Deng J., Qiu Y., Gao J., Li J., Guan L., Lee H., Zhou Q., Xiao J. (2021). Non-coding RNA basis of muscle atrophy. Mol. Ther. Nucleic Acids.

[8] Guerville F., Barreto P.D.S., Ader I., Andrieu S., Casteilla L., Dray C., Fazilleau N., Guyonnet S., Langin D., Liblau R. (2020). Revisiting the hallmarks of aging to identify markers of biological age. J. Prev. Alzheimer’s Dis..

[9] Carter H.N., Pauly M., Tryon L.D., Hood D.A. (2018). Effect of contractile activity on PGC-1α transcription in young and aged skeletal muscle. J. Appl. Physiol..

[10] Benayoun B., Pollina E.A., Brunet A. (2015). Epigenetic regulation of ageing: Linking environmental inputs to genomic stability. Nat. Rev. Mol. Cell Biol..

[11] Nagarajan P., Garcia P.A.A., Iyer C.C., Popova L.V., Arnold W.D., Parthun M.R. (2019). Early-onset aging and mitochondrial defects associated with loss of histone acetyltransferase 1 (Hat1). Aging Cell.

[12] Sorriento D., Di Vaia E., Iaccarino G. (2021). Physical Exercise: A Novel Tool to Protect Mitochondrial Health. Front. Physiol..

[13] McGee S.L., Hargreaves M. (2020). Exercise adaptations: Molecular mechanisms and potential targets for therapeutic benefit. Nat. Rev. Endocrinol..

[14] Kietzmann T., Petry A., Shvetsova A., Gerhold J., Görlach A. (2017). The epigenetic landscape related to reactive oxygen species formation in the cardiovascular system. Br. J. Pharmacol..

[15] Dimauro I., Paronetto M.P., Caporossi D. (2020). Exercise, redox homeostasis and the epigenetic landscape. Redox Biol..

[16] Kim H.K., Jeong Y.J., Song I.-S., Noh Y.H., Seo K.W., Kim M., Han J. (2017). Glucocorticoid receptor positively regulates transcription of FNDC5 in the liver. Sci. Rep..

[17] Liu C., Liu A.-S., Zhong D., Wang C.-G., Yu M., Zhang H.-W., Xiao H., Liu J.-H., Zhang J., Yin K. (2021). Circular RNA AFF4 modulates osteogenic differentiation in BM-MSCs by activating SMAD1/5 pathway through miR-135a-5p/FNDC5/Irisin axis. Cell Death Dis..

[18] Jia Y., Gao G., Song H., Cai D., Yang X., Zhao R. (2016). Low-protein diet fed to crossbred sows during pregnancy and lactation enhances myostatin gene expression through epigenetic regulation in skeletal muscle of weaning piglets. Eur. J. Nutr..

[19] Elsner V.R., Lovatel G.A., Moysés F., Bertoldi K., Spindler C., Cechinel L.R., Muotri A.R., Siqueira I.R. (2013). Exercise induces age-dependent changes on epigenetic parameters in rat hippocampus: A preliminary study. Exp. Gerontol..

[20] Palasz E., Wysocka A., Gasiorowska A., Chalimoniuk M., Niewiadomski W., Niewiadomska G. (2020). BDNF as a Promising Therapeutic Agent in Parkinson’s Disease. Int. J. Mol. Sci..

[21] Song J., Zhang Z., Dong Z., Liu X., Liu Y., Li X., Xu Y., Guo Y., Wang N., Zhang M. (2022). MicroRNA-122-5p Aggravates Angiotensin II-Mediated Myocardial Fibrosis and Dysfunction in Hypertensive Rats by Regulating the Elabela/Apelin-APJ and ACE2-GDF15-Porimin Signaling. J. Cardiovasc. Transl. Res..

[22] Tsika R.W., Herrick R.E., Baldwin K.M. (1987). Subunit composition of rodent isomyosins and their distribution in hindlimb skeletal muscles. J. Appl. Physiol..

[23] Smerdu V., Mizrachi I.K., Campione M., Leinwand L., Schiaffino S. (1994). Type IIx myosin heavy chain transcripts are expressed in type IIb fibers of human skeletal muscle. Am. J. Physiol. Physiol..

[24] Yan Z., Okutsu M., Akhtar Y.N., Lira V.A. (2011). Regulation of exercise-induced fiber type transformation, mitochondrial biogenesis, and angiogenesis in skeletal muscle. J. Appl. Physiol..

[25] Staron R.S., Hagerman F.C., Hikida R.S., Murray T.F., Hostler D.P., Crill M.T., Ragg K.E., Toma K. (2000). Fiber Type Composition of the Vastus Lateralis Muscle of Young Men and Women. J. Histochem. Cytochem..

[26] Bloemberg D., Quadrilatero J. (2012). Rapid Determination of Myosin Heavy Chain Expression in Rat, Mouse, and Human Skeletal Muscle Using Multicolor Immunofluorescence Analysis. PLoS ONE.

[27] D’Amico D., Gammazza A.M., Macaluso F., Paladino L., Scalia F., Spinoso G., Dimauro I., Caporossi D., Cappello F., Di Felice V. (2021). Sex-based differences after a single bout of exercise on PGC1α isoforms in skeletal muscle: A pilot study. FASEB J..

[28] Wallace D.C. (2005). A Mitochondrial Paradigm of Metabolic and Degenerative Diseases, Aging, and Cancer: A Dawn for Evolutionary Medicine. Annu. Rev. Genet..

[29] Bornstein R., Gonzalez B., Johnson S.C. (2020). Mitochondrial pathways in human health and aging. Mitochondrion.

[30] Zimmermann A., Madreiter-Sokolowski C., Stryeck S., Abdellatif M. (2021). Targeting the Mitochondria-Proteostasis Axis to Delay Aging. Front. Cell Dev. Biol..

[31] Garcia S., Nissanka N., Mareco E.A., Rossi S., Peralta S., Díaz F., Rotundo R.L., Carvalho R.F., Moraes C.T. (2018). Overexpression of PGC-1α in aging muscle enhances a subset of young-like molecular patterns. Aging Cell.

[32] Yang S., Loro E., Wada S., Kim B., Tseng W.-J., Li K., Khurana T.S., Arany Z. (2020). Functional effects of muscle PGC-1alpha in aged animals. Skelet. Muscle.

[33] Faitg J., Leduc-Gaudet J.-P., Reynaud O., Ferland G., Gaudreau P., Gouspillou G. (2019). Effects of Aging and Caloric Restriction on Fiber Type Composition, Mitochondrial Morphology and Dynamics in Rat Oxidative and Glycolytic Muscles. Front. Physiol..

[34] Touvier T., De Palma C., Rigamonti E., Scagliola A., Incerti E., Mazelin L., Thomas J.-L., D’Antonio M., Politi L.S., Schaeffer L. (2015). Muscle-specific Drp1 overexpression impairs skeletal muscle growth via translational attenuation. Cell Death Dis..

[35] Favaro G., Romanello V., Varanita T., Desbats M.A., Morbidoni V., Tezze C., Albiero M., Canato M., Gherardi G., De Stefani D. (2019). DRP1-mediated mitochondrial shape controls calcium homeostasis and muscle mass. Nat. Commun..

[36] Sebastián D., Sorianello E., Segalés J., Irazoki A., Ruiz-Bonilla V., Sala D., Planet E., Berenguer-Llergo A., Muñoz J.P., Sánchez-Feutrie M. (2016). Mfn2 deficiency links age-related sarcopenia and impaired autophagy to activation of an adaptive mitophagy pathway. EMBO J..

[37] Tezze C., Romanello V., Desbats M.A., Fadini G.P., Albiero M., Favaro G., Ciciliot S., Soriano M.E., Morbidoni V., Cerqua C. (2017). Age-Associated Loss of OPA1 in Muscle Impacts Muscle Mass, Metabolic Homeostasis, Systemic Inflammation, and Epithelial Senescence. Cell Metab..

[38] Beregi E., Regius O., Hüttl T., Göbl Z. (1988). Age-related changes in the skeletal muscle cells. Z. Gerontol..

[39] Beregi E., Regius O. (1987). Comparative morphological study of age related mitochondrial changes of the lymphocytes and skeletal muscle cells. Acta Morphol. Hung..

[40] Navratil M., Terman A., Arriaga E.A. (2008). Giant mitochondria do not fuse and exchange their contents with normal mitochondria. Exp. Cell Res..

[41] Taghizadeh G., Pourahmad J., Mehdizadeh H., Foroumadi A., Torkaman-Boutorabi A., Hassani S., Naserzadeh P., Shariatmadari R., Gholami M., Rouini M.R. (2016). Protective effects of physical exercise on MDMA-induced cognitive and mitochondrial impairment. Free Radic. Biol. Med..

[42] Flis D.J., Olek R.A., Kaczor J.J., Rodziewicz E., Halon M., Antosiewicz J., Wozniak M., Gabbianelli R., Ziolkowski W. (2016). Exercise-Induced Changes in Caveolin-1, Depletion of Mitochondrial Cholesterol, and the Inhibition of Mitochondrial Swelling in Rat Skeletal Muscle but Not in the Liver. Oxidative Med. Cell. Longev..

[43] Oliveira A.N., Richards B.J., Slavin M., Hood D.A. (2021). Exercise Is Muscle Mitochondrial Medicine. Exerc. Sport Sci. Rev..

[44] Carter H.N., Kim Y., Erlich A.T., Zarrin-Khat D., Hood D.A. (2018). Autophagy and mitophagy flux in young and aged skeletal muscle following chronic contractile activity. J. Physiol..

[45] Chen C.C.W., Erlich A.T., Crilly M.J., Hood D.A. (2018). Parkin is required for exercise-induced mitophagy in muscle: Impact of aging. Am. J. Physiol. Metab..

[46] Leduc-Gaudet J.-P., Reynaud O., Hussain S.N., Gouspillou G. (2019). Parkin overexpression protects from ageing-related loss of muscle mass and strength. J. Physiol..

[47] Gouspillou G., Godin R., Piquereau J., Picard M., Mofarrahi M., Mathew J., Purves-Smith F.M., Sgarioto N., Hepple R.T., Burelle Y. (2018). Protective role of Parkin in skeletal muscle contractile and mitochondrial function. J. Physiol..

[48] Ryu D., Mouchiroud L., Andreux P.A., Katsyuba E., Moullan N., Nicolet-Dit-Félix A.A., Williams E.G., Jha P., Lo Sasso G., Huzard D. (2016). Urolithin A induces mitophagy and prolongs lifespan in C. elegans and increases muscle function in rodents. Nat. Med..

[49] Martinus R.D., Garth G.P., Webster T.L., Cartwright P., Naylor D.J., Høj P.B., Hoogenraad N.J. (1996). Selective Induction of Mitochondrial Chaperones in Response to Loss of the Mitochondrial Genome. J. Biol. Inorg. Chem..

[50] Zhao Q., Wang J., Levichkin I.V., Stasinopoulos S., Ryan M., Hoogenraad N.J. (2002). A mitochondrial specific stress response in mammalian cells. EMBO J..

[51] Durieux J., Wolff S., Dillin A. (2011). The Cell-Non-Autonomous Nature of Electron Transport Chain-Mediated Longevity. Cell.

[52] Cordeiro A.V., Peruca G.F., Braga R.R., Brícola R.S., Lenhare L., Silva V.R.R., Anaruma C.P., Katashima C.K., Crisol B.M., Barbosa L.T. (2021). High-intensity exercise training induces mitonuclear imbalance and activates the mitochondrial unfolded protein response in the skeletal muscle of aged mice. GeroScience.

[53] Cordeiro A.V., Brícola R.S., Braga R.R., Lenhare L., Silva V.R.R., Anaruma C.P., Katashima C.K., Crisol B.M., Simabuco F.M., Silva A.S.R. (2020). Aerobic Exercise Training Induces the Mitonuclear Imbalance and UPRmt in the Skeletal Muscle of Aged Mice. J. Gerontol. Ser. A.

[54] Zhang H., Ryu D., Wu Y., Gariani K., Wang X., Luan P., D’Amico D., Ropelle E.R., Lutolf M.P., Aebersold R. (2016). NAD^+^ repletion improves mitochondrial and stem cell function and enhances life span in mice. Science.

[55] Liu X., Jiang N., Hughes B., Bigras E., Shoubridge E., Hekimi S. (2005). Evolutionary conservation of the *clk-1*-dependent mechanism of longevity: Loss of *mclk1* increases cellular fitness and lifespan in mice. Genes Dev..

[56] Houtkooper R., Mouchiroud L., Ryu D., Moullan N., Katsyuba E., Knott G.W., Williams R.W., Auwerx J. (2013). Mitonuclear protein imbalance as a conserved longevity mechanism. Nature.

[57] Jensen M.B., Jasper H. (2014). Mitochondrial Proteostasis in the Control of Aging and Longevity. Cell Metab..

[58] Auwerx J., Li T.Y. (2020). A conserved role of CBP/p300 in mitochondrial stress response and longevity. FASEB J..

[59] Wang N., Wang C., Zhao H., He Y., Lan B., Sun L., Gao Y. (2021). The MAMs Structure and Its Role in Cell Death. Cells.

[60] Csordás G., Renken C., Várnai P., Walter L., Weaver D., Buttle K.F., Balla T., Mannella C.A., Hajnóczky G. (2006). Structural and functional features and significance of the physical linkage between ER and mitochondria. J. Cell Biol..

[61] Yu H., Sun C., Gong Q., Feng D. (2021). Mitochondria-Associated Endoplasmic Reticulum Membranes in Breast Cancer. Front. Cell Dev. Biol..

[62] Silva-Palacios A., Zazueta C., Pedraza-Chaverri J. (2020). ER membranes associated with mitochondria: Possible therapeutic targets in heart-associated diseases. Pharmacol. Res..

[63] Jungbluth H., Treves S., Zorzato F., Sarkozy A., Ochala J., Sewry C., Phadke R., Gautel M., Muntoni F. (2018). Congenital myopathies: Disorders of excitation–contraction coupling and muscle contraction. Nat. Rev. Neurol..

[64] Cárdenas C., Miller R.A., Smith I., Bui T., Molgó J., Müller M., Vais H., Cheung K.-H., Yang J., Parker I. (2010). Essential Regulation of Cell Bioenergetics by Constitutive InsP3 Receptor Ca^2+^ Transfer to Mitochondria. Cell.

[65] Filippin L., Magalhães P.J., Di Benedetto G., Colella M., Pozzan T. (2003). Stable Interactions between Mitochondria and Endoplasmic Reticulum Allow Rapid Accumulation of Calcium in a Subpopulation of Mitochondria. J. Biol. Chem..

[66] Gherardi G., Monticelli H., Rizzuto R., Mammucari C. (2020). The Mitochondrial Ca^2+^ Uptake and the Fine-Tuning of Aerobic Metabolism. Front. Physiol..

[67] Friedman J.R., Lackner L.L., West M., DiBenedetto J.R., Nunnari J., Voeltz G.K. (2011). ER Tubules Mark Sites of Mitochondrial Division. Science.

[68] Saotome M., Safiulina D., Szabadkai G., Das S., Fransson A., Aspenstrom P., Rizzuto R., Hajnoczky G. (2008). Bidirectional Ca^2+^ dependent control of mitochondrial dynamics by the Miro GTPase. Proc. Natl. Acad. Sci. USA.

[69] Madreiter-Sokolowski C.T., Thomas C., Ristow M. (2020). Interrelation between ROS and Ca^2+^ in aging and age-related diseases. Redox Biol..

[70] Dong Z., Shanmughapriya S., Tomar D., Siddiqui N., Lynch S., Nemani N., Breves S.L., Zhang X., Tripathi A., Palaniappan P. (2017). Mitochondrial Ca^2+^ Uniporter Is a Mitochondrial Luminal Redox Sensor that Augments MCU Channel Activity. Mol. Cell.

[71] Joiner M.-L.A., Koval O.M., Li J., He B.J., Allamargot C., Gao Z., Luczak E.D., Hall D.D., Fink B.D., Chen B. (2012). CaMKII determines mitochondrial stress responses in heart. Nature.

[72] Ashkavand Z., Sarasija S., Ryan K.C., Laboy J.T., Norman K.R. (2020). Corrupted ER-mitochondrial calcium homeostasis promotes the collapse of proteostasis. Aging Cell.

[73] Cadenas E., Boveris A. (1980). Enhancement of hydrogen peroxide formation by protophores and ionophores in antimycin-supplemented mitochondria. Biochem. J..

[74] Brookes P., Yoon Y., Robotham J.L., Anders M.W., Sheu S.-S. (2004). Calcium, ATP, and ROS: A mitochondrial love-hate triangle. Am. J. Physiol. Physiol..

[75] Gil-Hernández A., Silva-Palacios A. (2020). Relevance of endoplasmic reticulum and mitochondria interactions in age-associated diseases. Ageing Res. Rev..

[76] Palikaras K., Lionaki E., Tavernarakis N. (2015). Coordination of mitophagy and mitochondrial biogenesis during ageing in *C. elegans*. Nature.

[77] Ziegler D.V., Vindrieux D., Goehrig D., Jaber S., Collin G., Griveau A., Wiel C., Bendridi N., Djebali S., Farfariello V. (2021). Calcium channel ITPR2 and mitochondria–ER contacts promote cellular senescence and aging. Nat. Commun..

[78] Müller M., Ahumada-Castro U., Sanhueza M., Gonzalez-Billault C., Felipe A., Court F.A., Cárdenas C. (2018). Mitochondria and Calcium Regulation as Basis of Neurodegeneration Associated with Aging. Front. Neurosci..

[79] Cherubini M., Lopez-Molina L., Gines S. (2020). Mitochondrial fission in Huntington’s disease mouse striatum disrupts ER-mitochondria contacts leading to disturbances in Ca^2+^ efflux and Reactive Oxygen Species (ROS) homeostasis. Neurobiol. Dis..

[80] Merle A., Jollet M., Britto F.A., Goustard B., Bendridi N., Rieusset J., Ollendorff V., Favier F.B. (2019). Endurance exercise decreases protein synthesis and ER-mitochondria contacts in mouse skeletal muscle. J. Appl. Physiol..

[81] Flis D.J., Dzik K., Kaczor J.J., Halon-Golabek M., Antosiewicz J., Wieckowski M.R., Ziolkowski W. (2018). Swim Training Modulates Skeletal Muscle Energy Metabolism, Oxidative Stress, and Mitochondrial Cholesterol Content in Amyotrophic Lateral Sclerosis Mice. Oxidative Med. Cell. Longev..

[82] Cheng A.J., Place N., Westerblad H. (2018). Molecular Basis for Exercise-Induced Fatigue: The Importance of Strictly Controlled Cellular Ca^2+^ Handling. Cold Spring Harb. Perspect. Med..

[83] Patergnani S., Suski J.M., Agnoletto C., Bononi A., Bonora M., De Marchi E., Giorgi C., Marchi S., Missiroli S., Poletti F. (2011). Calcium signaling around Mitochondria Associated Membranes (MAMs). Cell Commun. Signal..

[84] Rizzuto R., Marchi S., Bonora M., Aguiari P., Bononi A., De Stefani D., Giorgi C., Leo S., Rimessi A., Siviero R. (2009). Ca^2+^ transfer from the ER to mitochondria: When, how and why. Biochim. Biophys. Acta.

[85] Talens R.P., Christensen K., Putter H., Willemsen G., Christiansen L., Kremer D., Suchiman H.E.D., Slagboom P., Boomsma D.I., Heijmans B.T. (2012). Epigenetic variation during the adult lifespan: Cross-sectional and longitudinal data on monozygotic twin pairs. Aging Cell.

[86] Turner D.C., Gorski P.P., Maasar M.F., Seaborne R.A., Baumert P., Brown A.D., Kitchen M.O., Erskine R.M., Dos-Remedios I., Voisin S. (2020). DNA methylation across the genome in aged human skeletal muscle tissue and muscle-derived cells: The role of HOX genes and physical activity. Sci. Rep..

[87] Sailani M.R., Halling J.F., Møller H.D., Lee H., Plomgaard P., Pilegaard H., Snyder M.P., Regenberg B. (2019). Lifelong physical activity is associated with promoter hypomethylation of genes involved in metabolism, myogenesis, contractile properties and oxidative stress resistance in aged human skeletal muscle. Sci. Rep..

[88] Horvath S., Raj K. (2018). DNA methylation-based biomarkers and the epigenetic clock theory of ageing. Nat. Rev. Genet..

[89] Davegårdh C., Wedin E.H., Broholm C., Henriksen T., Pedersen M., Pedersen B.K., Scheele C., Ling C. (2019). Sex influences DNA methylation and gene expression in human skeletal muscle myoblasts and myotubes. Stem Cell Res. Ther..

[90] Koczor C.A., Ludlow I., Fields E.J., Jiao Z., Ludaway T., Russ R., Lewis W. (2016). Mitochondrial polymerase gamma dysfunction and aging cause cardiac nuclear DNA methylation changes. Physiol. Genom..

[91] Rawat P.S., Jaiswal A., Khurana A., Bhatti J.S., Navik U. (2021). Doxorubicin-induced cardiotoxicity: An update on the molecular mechanism and novel therapeutic strategies for effective management. Biomed. Pharmacother..

[92] Hua X.-M., Wang J., Qian D.-M., Song J.-Y., Chen H., Zhu X.-L., Zhou R., Zhao Y.-D., Zhou X.-Z., Li L. (2015). DNA methylation level of promoter region of activating transcription factor 5 in glioma. J. Zhejiang Univ. Sci. B.

[93] Gao F., Xia Y., Wang J., Lin Z., Ou Y., Liu X., Liu W., Zhou B., Luo H., Zhou B. (2014). Integrated analyses of DNA methylation and hydroxymethylation reveal tumor suppressive roles of ECM1, ATF5, and EOMESin human hepatocellular carcinoma. Genome Biol..

[94] Tian Y., Garcia G., Bian Q., Steffen K.K., Joe L., Wolff S., Meyer B.J., Dillin A. (2016). Mitochondrial Stress Induces Chromatin Reorganization to Promote Longevity and UPR^mt^. Cell.

[95] Merkwirth C., Jovaisaite V., Durieux J., Matilainen O., Jordan S.D., Quiros P.M., Steffen K.K., Williams E.G., Mouchiroud L., Tronnes S.U. (2016). Two Conserved Histone Demethylases Regulate Mitochondrial Stress-Induced Longevity. Cell.

[96] Li T.Y., Sleiman M.B., Li H., Gao A.W., Mottis A., Bachmann A.M., El Alam G., Li X., Goeminne L.J.E., Schoonjans K. (2021). The transcriptional coactivator CBP/p300 is an evolutionarily conserved node that promotes longevity in response to mitochondrial stress. Nat. Aging.

[97] Shao L.-W., Peng Q., Dong M., Gao K., Li Y., Li Y., Li C.-Y., Liu Y. (2020). Histone deacetylase HDA-1 modulates mitochondrial stress response and longevity. Nat. Commun..

[98] Drummond M.J., McCarthy J.J., Fry C.S., Esser K.A., Rasmussen B.B. (2008). Aging differentially affects human skeletal muscle microRNA expression at rest and after an anabolic stimulus of resistance exercise and essential amino acids. Am. J. Physiol. Metab..

[99] Jia B., Liu Y., Li Q., Zhang J., Ge C., Wang G., Chen G., Liu D., Yang F. (2020). Altered miRNA and mRNA Expression in Sika Deer Skeletal Muscle with Age. Genes.

[100] Enielsen S., Ehvid T., Ekelly M., Elindegaard B., Edethlefsen C., Ewinding K., Emathur N., Escheele C., Pedersen B.K., Laye M.J. (2013). Muscle specific miRNAs are induced by testosterone and independently upregulated by age. Front. Physiol..

[101] Nie Y., Sato Y., Wang C., Yue F., Kuang S., Gavin T.P. (2016). Impaired exercise tolerance, mitochondrial biogenesis, and muscle fiber maintenance in miR-133a–deficient mice. FASEB J..

[102] Dahlmans D., Houzelle A., Andreux P., Wang X., Jörgensen J.A., Moullan N., Daemen S., Kersten S., Auwerx J., Hoeks J. (2019). MicroRNA-382 silencing induces a mitonuclear protein imbalance and activates the mitochondrial unfolded protein response in muscle cells. J. Cell. Physiol..

[103] Morelli M., Wang X., Matarese A., Chavez C., Santulli G. (2019). Dual Microrna-Targeting Rescues the Impaired Mitochondrial Unfolded Protein Response in Heart Failure. Circulation.

[104] Barres R., Yan J., Egan B., Treebak J.T., Rasmussen M., Fritz T., Caidahl K., Krook A., O’Gorman D.J., Zierath J.R. (2012). Acute Exercise Remodels Promoter Methylation in Human Skeletal Muscle. Cell Metab..

[105] Bajpeyi S., Covington J.D., Taylor E.M., Stewart L.K., Galgani J.E., Henagan T.M. (2017). Skeletal Muscle PGC1α −1 Nucleosome Position and −260 nt DNA Methylation Determine Exercise Response and Prevent Ectopic Lipid Accumulation in Men. Endocrinology.

[106] Hunter D.J., James L., Hussey B., Wadley A.J., Lindley M.R., Mastana S.S. (2019). Impact of aerobic exercise and fatty acid supplementation on global and gene-specific DNA methylation. Epigenetics.

[107] Landen S., Voisin S., Craig J.M., McGee S.L., Lamon S., Eynon N. (2019). Genetic and epigenetic sex-specific adaptations to endurance exercise. Epigenetics.

[108] Brown W. (2015). Exercise-associated DNA methylation change in skeletal muscle and the importance of imprinted genes: A bioinformatics meta-analysis. Br. J. Sports Med..

[109] Maasar M.-F., Turner D.C., Gorski P.P., Seaborne R.A., Strauss J.A., Shepherd S.O., Cocks M., Pillon N.J., Zierath J.R., Hulton A.T. (2021). The Comparative Methylome and Transcriptome After Change of Direction Compared to Straight Line Running Exercise in Human Skeletal Muscle. Front. Physiol..

[110] Rasmussen M., Zierath J., Barrès R. (2014). Dynamic epigenetic responses to muscle contraction. Drug Discov. Today.

[111] Small L., Ingerslev L.R., Manitta E., Laker R.C., Hansen A.N., Deeney B., Carrié A., Couvert P., Barrès R. (2021). Ablation of DNA-methyltransferase 3A in skeletal muscle does not affect energy metabolism or exercise capacity. PLoS Genet..

[112] Lochmann T.L., Thomas R.R., Bennett J.P., Taylor S.M. (2015). Epigenetic Modifications of the PGC-1α Promoter during Exercise Induced Expression in Mice. PLoS ONE.

[113] Joseph J.S., Anand K., Malindisa S.T., Oladipo A.O., Fagbohun O.F. (2021). Exercise, CaMKII, and type 2 diabetes. EXCLI J..

[114] Joseph J.S., Ayeleso A.O., Mukwevho E. (2017). Exercise increases hyper-acetylation of histones on the Cis -element of NRF-1 binding to the Mef2a promoter: Implications on type 2 diabetes. Biochem. Biophys. Res. Commun..

[115] Czubryt M.P., McAnally J., Fishman G.I., Olson E.N. (2003). Regulation of peroxisome proliferator-activated receptor γ coactivator 1α (PGC-1α) and mitochondrial function by MEF2 and HDAC5. Proc. Natl. Acad. Sci. USA.

[116] Masuzawa R., Konno R., Ohsawa I., Watanabe A., Kawano F. (2018). Muscle type-specific RNA polymerase II recruitment during PGC-1α gene transcription after acute exercise in adult rats. J. Appl. Physiol..

[117] Jacques M., Hiam D., Craig J., Barrès R., Eynon N., Voisin S. (2019). Epigenetic changes in healthy human skeletal muscle following exercise—A systematic review. Epigenetics.

[118] Russell A.P., Lamon S., Boon H., Wada S., Güller I., Brown E.L., Chibalin A.V., Zierath J.R., Snow R.J., Stepto N.I. (2013). Regulation of miRNAs in human skeletal muscle following acute endurance exercise and short-term endurance training. J. Physiol..

[119] Rodrigues A.C., Spagnol A.R., Frias F.D.T., de Mendonça M., Araújo H.N., Guimarães D., Silva W.J., Bolin A.P., Murata G.M., Silveira L. (2021). Intramuscular Injection of miR-1 Reduces Insulin Resistance in Obese Mice. Front. Physiol..

[120] Sun Y., Cui D., Zhang Z., Zhang Q., Ji L., Ding S. (2016). Voluntary wheel exercise alters the levels of miR-494 and miR-696 in the skeletal muscle of C57BL/6 mice. Comp. Biochem. Physiol. Part B Biochem. Mol. Biol..

[121] Massart J., Sjögren R.J.O., Egan B., Garde C., Lindgren M., Gu W., Ferreira D.M.S., Katayama M., Ruas J.L., Barrès R. (2021). Endurance exercise training-responsive miR-19b-3p improves skeletal muscle glucose metabolism. Nat. Commun..

[122] Hicks S.D., Jacob P., Middleton F.A., Pérez O., Gagnon Z. (2018). Distance running alters peripheral microRNAs implicated in metabolism, fluid balance, and myosin regulation in a sex-specific manner. Physiol. Genom..

[123] Niu Y., DesMarais T.L., Tong Z., Yao Y., Costa M. (2015). Oxidative stress alters global histone modification and DNA methylation. Free Radic. Biol. Med..

[124] Pradhan N., Parbin S., Kar S., Das L., Kirtana R., Seshadri G.S., Sengupta D., Deb M., Kausar C., Patra S.K. (2019). Epigenetic silencing of genes enhanced by collective role of reactive oxygen species and MAPK signaling downstream ERK/Snail axis: Ectopic application of hydrogen peroxide repress CDH1 gene by enhanced DNA methyltransferase activity in human breast cancer. Biochim. Biophys. Acta (BBA) Mol. Basis Dis..

[125] Powers S.K., Deminice R., Ozdemir M., Yoshihara T., Bomkamp M.P., Hyatt H. (2020). Exercise-induced oxidative stress: Friend or foe?. J. Sport Health Sci..

[126] Rosini E., Pollegioni L. (2022). Reactive oxygen species as a double-edged sword: The role of oxidative enzymes in antitumor therapy. BioFactors.

[127] Zeng M.Y., Miralda I., Armstrong C.L., Uriarte S.M., Bagaitkar J. (2019). The roles of NADPH oxidase in modulating neutrophil effector responses. Mol. Oral Microbiol..

[128] Taherkhani S., Suzuki K., Castell L. (2020). A Short Overview of Changes in Inflammatory Cytokines and Oxidative Stress in Response to Physical Activity and Antioxidant Supplementation. Antioxidants.

[129] Ristow M., Zarse K., Oberbach A., Klöting N., Birringer M., Kiehntopf M., Stumvoll M., Kahn C.R., Blüher M. (2009). Antioxidants prevent health-promoting effects of physical exercise in humans. Proc. Natl. Acad. Sci. USA.

[130] Wang Z., Bo H., Song Y., Li C., Zhang Y. (2022). Mitochondrial ROS Produced by Skeletal Muscle Mitochondria Promote the Decisive Signal for UPRmt Activation. BioMed Res. Int..

[131] Thirupathi A., Pinho R.A., Chang Y.-Z. (2020). Physical exercise: An inducer of positive oxidative stress in skeletal muscle aging. Life Sci..

[132] Nilsson M.I., Tarnopolsky M.A. (2019). Mitochondria and Aging—The Role of Exercise as a Countermeasure. Biology.

[133] Tomasetti M., Gaetani S., Monaco F., Neuzil J., Santarelli L. (2019). Epigenetic Regulation of miRNA Expression in Malignant Mesothelioma: miRNAs as Biomarkers of Early Diagnosis and Therapy. Front. Oncol..

[134] Zhou X., Zhuang Z., Wang W., He L., Wu H., Cao Y., Pan F., Zhao J., Hu Z., Sekhar C. (2016). OGG1 is essential in oxidative stress induced DNA demethylation. Cell. Signal..

[135] Le D.D., Fujimori D.G. (2012). Protein and nucleic acid methylating enzymes: Mechanisms and regulation. Curr. Opin. Chem. Biol..

[136] Coulter J.B., O’Driscoll C.M., Bressler J.P. (2013). Hydroquinone Increases 5-Hydroxymethylcytosine Formation through Ten Eleven Translocation 1 (TET1) 5-Methylcytosine Dioxygenase. J. Biol. Chem..

[137] García-Giménez J.L., Romá-Mateo C., Pérez-Machado G., Peiró-Chova L., Pallardó F.V. (2017). Role of glutathione in the regulation of epigenetic mechanisms in disease. Free Radic. Biol. Med..

[138] Thirupathi A., De Souza C.T. (2017). Multi-regulatory network of ROS: The interconnection of ROS, PGC-1 alpha, and AMPK-SIRT1 during exercise. J. Physiol. Biochem..

[139] Stephens N.A., Brouwers B., Eroshkin A.M., Yi F., Cornnell H.H., Meyer C., Goodpaster B.H., Pratley R.E., Smith S.R., Sparks L.M. (2018). Exercise Response Variations in Skeletal Muscle PCr Recovery Rate and Insulin Sensitivity Relate to Muscle Epigenomic Profiles in Individuals With Type 2 Diabetes. Diabetes Care.

[140] Seo E., Kang H., Choi H., Choi W., Jun H.-S. (2019). Reactive oxygen species-induced changes in glucose and lipid metabolism contribute to the accumulation of cholesterol in the liver during aging. Aging Cell.

[141] Marmisolle I., Martínez J., Liu J., Mastrogiovanni M., Fergusson M.M., Rovira I.I., Castro L., Trostchansky A., Moreno M., Cao L. (2017). Reciprocal regulation of acetyl-CoA carboxylase 1 and senescence in human fibroblasts involves oxidant mediated p38 MAPK activation. Arch. Biochem. Biophys..

[142] Morales-Alamo D., Guerra B., Ponce-González J.G., Guadalupe-Grau A., Santana A., Martin-Rincon M., Gelabert-Rebato M., Cadefau J.A., Cusso R., Dorado C. (2017). Skeletal muscle signaling, metabolism, and performance during sprint exercise in severe acute hypoxia after the ingestion of antioxidants. J. Appl. Physiol..

[143] Miotto P.M., Holloway G.P. (2019). Exercise-induced reductions in mitochondrial ADP sensitivity contribute to the induction of gene expression and mitochondrial biogenesis through enhanced mitochondrial H_2_O_2_ emission. Mitochondrion.

[144] McGee S.L., Hargreaves M. (2019). Epigenetics and Exercise. Trends Endocrinol. Metab..

[145] Elliott P.J., Jirousek M. (2008). Sirtuins: Novel targets for metabolic disease. Curr. Opin. Investig. Drugs.

[146] Yang Y., Fu W., Chen J., Olashaw N., Zhang X., Nicosia S.V., Bhalla K., Bai W. (2007). SIRT1 sumoylation regulates its deacetylase activity and cellular response to genotoxic stress. Nat. Cell Biol..

[147] Zhang Y., Sun Z., Jia J., Du T., Zhang N., Tang Y., Fang Y., Fang D. (2021). Overview of Histone Modification. Adv. Exp. Med. Biol..

[148] Wang J.-X., Gao J., Ding S.-L., Wang K., Jiao J.-Q., Wang Y., Sun T., Zhou L.-Y., Long B., Zhang X.-J. (2015). Oxidative Modification of miR-184 Enables It to Target Bcl-xL and Bcl-w. Mol. Cell.

[149] Seok H., Lee H., Lee S., Ahn S.H., Lee H.-S., Kim G.-W.D., Peak J., Park J., Cho Y.K., Jeong Y. (2020). Position-specific oxidation of miR-1 encodes cardiac hypertrophy. Nature.

[150] Natarelli L., Weber C. (2022). A Non-Canonical Link between Non-Coding RNAs and Cardiovascular Diseases. Biomedicines.

[151] Emde A., Hornstein E. (2014). mi RNA s at the interface of cellular stress and disease. EMBO J..

[152] Druz A., Betenbaugh M., Shiloach J. (2012). Glucose depletion activates mmu-miR-466h-5p expression through oxidative stress and inhibition of histone deacetylation. Nucleic Acids Res..

[153] He J., Xu Q., Jing Y., Agani F., Qian X., Carpenter R., Li Q., Wang X.-R., Peiper S.S., Lu Z. (2012). Reactive oxygen species regulate ERBB2 and ERBB3 expression via miR-199a/125b and DNA methylation. EMBO Rep..

[154] Safdar A., Abadi A., Akhtar M., Hettinga B.P., Tarnopolsky M.A. (2009). miRNA in the Regulation of Skeletal Muscle Adaptation to Acute Endurance Exercise in C57Bl/6J Male Mice. PLoS ONE.

[155] Pedersen B.K., Fischer C. (2007). Beneficial health effects of exercise—The role of IL-6 as a myokine. Trends Pharmacol. Sci..

[156] Pedersen B.K. (2013). Muscle as a Secretory Organ. Compr. Physiol..

[157] Pedersen B.K. (2011). Muscles and their myokines. J. Exp. Biol..

[158] Chow L.S., Gerszten R.E., Taylor J.M., Pedersen B.K., van Praag H., Trappe S., Febbraio M.A., Galis Z.S., Gao Y., Haus J.M. (2022). Exerkines in health, resilience and disease. Nat. Rev. Endocrinol..

[159] Bo H., Jiang N., Zhang Z.-Y., Ji L.-L., Zhang Y. (2014). Exercise and health: From evaluation of health-promoting effects of exercise to exploration of exercise mimetics. Sheng Li Ke Xue Jin Zhan [Prog. Physiol.].

[160] (2015). Exercise Metabolism. Cell Metab..

[161] Pedersen B.K., Febbraio M.A. (2008). Muscle as an Endocrine Organ: Focus on Muscle-Derived Interleukin. Physiol. Rev..

[162] Mangano G.D., Fouani M., D’Amico D., Di Felice V., Barone R. (2022). Cancer-Related Cachexia: The Vicious Circle between Inflammatory Cytokines, Skeletal Muscle, Lipid Metabolism and the Possible Role of Physical Training. Int. J. Mol. Sci..

[163] Nara H., Watanabe R. (2021). Anti-Inflammatory Effect of Muscle-Derived Interleukin-6 and Its Involvement in Lipid Metabolism. Int. J. Mol. Sci..

[164] Daou H.N. (2020). Exercise as an anti-inflammatory therapy for cancer cachexia: A focus on interleukin-6 regulation. Am. J. Physiol. Integr. Comp. Physiol..

[165] Pedersen B.K. (2017). Anti-inflammatory effects of exercise: Role in diabetes and cardiovascular disease. Eur. J. Clin. Investig..

[166] Padilha C.S., Borges F.H., Da Silva L.E.C.M., Frajacomo F.T.T., Jordao A.A., Duarte J.A., Cecchini R., Guarnier F.A., Deminice R. (2017). Resistance exercise attenuates skeletal muscle oxidative stress, systemic pro-inflammatory state, and cachexia in Walker-256 tumor-bearing rats. Appl. Physiol. Nutr. Metab..

[167] Santos J.D.M.B.D., Bachi A.L.L., Junior L.A.L., Foster R., Sierra A.P.R., Benetti M., Araújo J.R., Ghorayeb N., Kiss M.A.P.D., Vieira R.P. (2020). The Relationship of IL-8 and IL-10 Myokines and Performance in Male Marathon Runners Presenting Exercise-Induced Bronchoconstriction. Int. J. Environ. Res. Public Health.

[168] Li L., Mühlfeld C., Niemann B., Pan R., Li R., Hilfiker-Kleiner D., Chen Y., Rohrbach S. (2011). Mitochondrial biogenesis and PGC-1α deacetylation by chronic treadmill exercise: Differential response in cardiac and skeletal muscle. Basic Res. Cardiol..

[169] Boström P., Wu J., Jedrychowski M.P., Korde A., Ye L., Lo J.C., Rasbach K.A., Boström E.A., Choi J.H., Long J.Z. (2012). A PGC1-α-dependent myokine that drives brown-fat-like development of white fat and thermogenesis. Nature.

[170] Zhang R., Fu T., Zhao X., Qiu Y., Hu X., Shi H., Yin X. (2020). Association of Circulating Irisin Levels with Adiposity and Glucose Metabolic Profiles in a Middle-Aged Chinese Population: A Cross-Sectional Study. Diabetes Metab. Syndr. Obesity Targets Ther..

[171] Planella-Farrugia C., Comas F., Sabater-Masdeu M., Moreno M., Moreno-Navarrete J.M., Rovira O., Ricart W., Fernández-Real J.M. (2019). Circulating Irisin and Myostatin as Markers of Muscle Strength and Physical Condition in Elderly Subjects. Front. Physiol..

[172] Miyamoto-Mikami E., Sato K., Kurihara T., Hasegawa N., Fujie S., Fujita S., Sanada K., Hamaoka T., Tabata I., Iemitsu M. (2015). Endurance Training-Induced Increase in Circulating Irisin Levels Is Associated with Reduction of Abdominal Visceral Fat in Middle-Aged and Older Adults. PLoS ONE.

[173] Kim H.-J., So B., Choi M., Kang D., Song W. (2015). Resistance exercise training increases the expression of irisin concomitant with improvement of muscle function in aging mice and humans. Exp. Gerontol..

[174] Amanat S., Sinaei E., Panji M., MohammadporHodki R., Bagheri-Hosseinabadi Z., Asadimehr H., Fararouei M., Dianatinasab A. (2020). A Randomized Controlled Trial on the Effects of 12 Weeks of Aerobic, Resistance, and Combined Exercises Training on the Serum Levels of Nesfatin-1, Irisin-1 and HOMA-IR. Front. Physiol..

[175] Belviranlı M., Okudan N. (2018). Exercise training increases cardiac, hepatic and circulating levels of brain-derived neurotrophic factor and irisin in young and aged rats. Horm. Mol. Biol. Clin. Investig..

[176] Tsuchiya Y., Ando D., Takamatsu K., Goto K. (2015). Resistance exercise induces a greater irisin response than endurance exercise. Metabolism.

[177] He W., Wang P., Chen Q., Li C. (2020). Exercise enhances mitochondrial fission and mitophagy to improve myopathy following critical limb ischemia in elderly mice via the PGC1a/FNDC5/irisin pathway. Skelet. Muscle.

[178] Gomes M.J., Martinez P.F., Pagan L.U., Damatto R.L., Cezar M.D.M., Lima A.R.R., Okoshi K., Okoshi M.P. (2017). Skeletal muscle aging: Influence of oxidative stress and physical exercise. Oncotarget.

[179] McPherron A., Lawler A.M., Lee S.-J. (1997). Regulation of skeletal muscle mass in mice by a new TGF-p superfamily member. Nature.

[180] Shabkhiz F., Khalafi M., Rosenkranz S., Karimi P., Moghadami K. (2021). Resistance training attenuates circulating FGF-21 and myostatin and improves insulin resistance in elderly men with and without type 2 diabetes mellitus: A randomised controlled clinical trial. Eur. J. Sport Sci..

[181] Ryan A., Li G., Blumenthal J., Ortmeyer H. (2013). Aerobic exercise + weight loss decreases skeletal muscle myostatin expression and improves insulin sensitivity in older adults. Obesity.

[182] Jerobin J., Ramanjaneya M., Bettahi I., Parammal R., Siveen K.S., Alkasem M., Aye M., Sathyapalan T., Skarulis M., Atkin S.L. (2021). Regulation of circulating CTRP-2/CTRP-9 and GDF-8/GDF-15 by intralipids and insulin in healthy control and polycystic ovary syndrome women following chronic exercise training. Lipids Health Dis..

[183] Tan Z., Zhao M., Li J., Li S., Zhu S., Yao X., Gao X., Yang S. (2022). Myostatin is involved in skeletal muscle dysfunction in chronic obstructive pulmonary disease via Drp-1 mediated abnormal mitochondrial division. Ann. Transl. Med..

[184] Elia A., Cannavo A., Gambino G., Cimini M., Ferrara N., Kishore R., Paolocci N., Rengo G. (2021). Aging is associated with cardiac autonomic nerve fiber depletion and reduced cardiac and circulating BDNF levels. J. Geriatr. Cardiol..

[185] Belviranlı M., Okudan N. (2018). Exercise Training Protects Against Aging-Induced Cognitive Dysfunction via Activation of the Hippocampal PGC-1α/FNDC5/BDNF Pathway. NeuroMolecular Med..

[186] Matthews V.B., Åström M.-B., Chan S., Bruce C., Krabbe K.S., Prelovsek O., Åkerström T., Yfanti C., Broholm C., Mortensen O.H. (2009). Brain-derived neurotrophic factor is produced by skeletal muscle cells in response to contraction and enhances fat oxidation via activation of AMP-activated protein kinase. Diabetologia.

[187] Yang X., Brobst D., Chan W.S., Tse M.C.L., Herlea-Pana O., Ahuja P., Bi X., Zaw A.M., Kwong Z.S.W., Jia W.-H. (2019). Muscle-generated BDNF is a sexually dimorphic myokine that controls metabolic flexibility. Sci. Signal..

[188] Vinel C., Lukjanenko L., Batut A., Deleruyelle S., Pradère J.-P., Le Gonidec S., Dortignac A., Geoffre N., Pereira O., Karaz S. (2018). The exerkine apelin reverses age-associated sarcopenia. Nat. Med..

[189] Dundar A., Kocahan S., Sahin L. (2021). Associations of apelin, leptin, irisin, ghrelin, insulin, glucose levels, and lipid parameters with physical activity during eight weeks of regular exercise training. Arch. Physiol. Biochem..

[190] Son J.S., Chae S.A., Wang H., Chen Y., Iniguez A.B., de Avila J.M., Jiang Z., Zhu M.-J., Du M. (2020). Maternal Inactivity Programs Skeletal Muscle Dysfunction in Offspring Mice by Attenuating Apelin Signaling and Mitochondrial Biogenesis. Cell Rep..

[191] Coco M., Perciavalle V., Cavallari P., Bolzoni F., Graziano A.C.E., Perciavalle V. (2017). Effects of age and sex on epigenetic modification induced by an acute physical exercise. Medicine.

[192] Klymenko O., Brecklinghaus T., Dille M., Springer C., de Wendt C., Altenhofen D., Binsch C., Knebel B., Scheller J., Hardt C. (2020). Histone deacetylase 5 regulates interleukin 6 secretion and insulin action in skeletal muscle. Mol. Metab..

[193] Li D., Wang P., Wei W., Wang C., Zhong Y., Lv L., Wang J. (2021). Serum MicroRNA Expression Patterns in Subjects after the 5-km Exercise Are Strongly Associated with Cardiovascular Adaptation. Front. Physiol..

[194] Dorhoi A., Iannaccone M., Farinacci M., Faé K.C., Schreiber J., Moura-Alves P., Nouailles G., Mollenkopf H.-J., Oberbeck-Müller D., Jörg S. (2013). MicroRNA-223 controls susceptibility to tuberculosis by regulating lung neutrophil recruitment. J. Clin. Investig..

[195] Li M., He Y., Zhou Z., Ramirez T., Gao Y., Gao Y., Ross R.A., Cao H., Cai Y., Xu M. (2017). MicroRNA-223 ameliorates alcoholic liver injury by inhibiting the IL-6–p47phox–oxidative stress pathway in neutrophils. Gut.

[196] Ye C., Tong Y., Wu N., Wan G.-W., Zheng F., Chen J.-Y., Lei J.-Z., Zhou H., Chen A.-D., Wang J.-J. (2021). Inhibition of miR-135a-5p attenuates vascular smooth muscle cell proliferation and vascular remodeling in hypertensive rats. Acta Pharmacol. Sin..

[197] Espinal M.P., Gasperini C., Marzi M., Braccia C., Armirotti A., Pötzsch A., Walker T.L., Fabel K., Nicassio F., Kempermann G. (2019). MiR-135a-5p Is Critical for Exercise-Induced Adult Neurogenesis. Stem Cell Rep..

[198] Fan H., Zhang R., Tesfaye D., Tholen E., Looft C., Hölker M., Schellander K., Cinar M.U. (2012). Sulforaphane causes a major epigenetic repression of myostatin in porcine satellite cells. Epigenetics.

[199] Roberts T.C., EL Andaloussi S., Morris K.V., McClorey G., Wood M.J. (2012). Small RNA-Mediated Epigenetic Myostatin Silencing. Mol. Ther. Nucleic Acids.

[200] Zarfeshani A., Ngo S., Sheppard A.M. (2014). Leucine alters hepatic glucose/lipid homeostasis via the myostatin-AMP-activated protein kinase pathway—Potential implications for nonalcoholic fatty liver disease. Clin. Epigenetics.

[201] Drummond M.J., Glynn E.L., Fry C.S., Dhanani S., Volpi E., Rasmussen B.B. (2009). Essential Amino Acids Increase MicroRNA-499, -208b, and -23a and Downregulate Myostatin and Myocyte Enhancer Factor 2C mRNA Expression in Human Skeletal Muscle. J. Nutr..

[202] Tomiga Y., Sakai K., Ra S., Kusano M., Ito A., Uehara Y., Takahashi H., Kawanaka K., Soejima H., Higaki Y. (2021). Short-term running exercise alters DNA methylation patterns in neuronal nitric oxide synthase and brain-derived neurotrophic factor genes in the mouse hippocampus and reduces anxiety-like behaviors. FASEB J..

[203] Cechinel L.R., Basso C.G., Bertoldi K., Schallenberger B., de Meireles L.C.F., Siqueira I.R. (2016). Treadmill exercise induces age and protocol-dependent epigenetic changes in prefrontal cortex of Wistar rats. Behav. Brain Res..

[204] Gomez-Pinilla F., Zhuang Y., Feng J., Ying Z., Fan G. (2011). Exercise impacts brain-derived neurotrophic factor plasticity by engaging mechanisms of epigenetic regulation. Eur. J. Neurosci..

[205] Sleiman S.F., Henry J., Al-Haddad R., El Hayek L., Abou Haidar E., Stringer T., Ulja D., Karuppagounder S.S., Holson E.B., Ratan R.R. (2016). Exercise promotes the expression of brain derived neurotrophic factor (BDNF) through the action of the ketone body β-hydroxybutyrate. eLife.

[206] Tu Z., Li Y., Dai Y., Li L., Lv G., Chen I., Wang B. (2017). MiR-140/BDNF axis regulates normal human astrocyte proliferation and LPS-induced IL-6 and TNF-α secretion. Biomed. Pharmacother..

[207] Zhang K., Wu S., Li Z., Zhou J. (2017). MicroRNA-211/BDNF axis regulates LPS-induced proliferation of normal human astrocyte through PI3K/AKT pathway. Biosci. Rep..

[208] Zheng P., Bin H., Chen W. (2019). Inhibition of microRNA-103a inhibits the activation of astrocytes in hippocampus tissues and improves the pathological injury of neurons of epilepsy rats by regulating BDNF. Cancer Cell Int..

[209] Bao T.-H., Miao W., Han J.-H., Yin M., Yan Y., Wang W.-W., Zhu Y.-H. (2014). Spontaneous Running Wheel Improves Cognitive Functions of Mouse Associated with miRNA Expressional Alteration in Hippocampus Following Traumatic Brain Injury. J. Mol. Neurosci..

[210] Mishra A., Kohli S., Dua S., Thinlas T., Mohammad G., Pasha M.A.Q. (2015). Genetic differences and aberrant methylation in the apelin system predict the risk of high-altitude pulmonary edema. Proc. Natl. Acad. Sci. USA.

[211] Miller C.N., Dye J.A., Schladweiler M.C., Richards J.H., Ledbetter A.D., Stewart E., Kodavanti U.P. (2018). Acute inhalation of ozone induces DNA methylation of apelin in lungs of Long-Evans rats. Inhal. Toxicol..

[212] Keleher M.R., Zaidi S., Shah S., Oakley M.E., Pavlatos C., El Idrissi S., Xing X., Li D., Wang T., Cheverud J.M. (2018). Maternal high-fat diet associated with altered gene expression, DNA methylation, and obesity risk in mouse offspring. PLoS ONE.

[213] Wan Y., Zeng Z.-C., Xi M., Wan S., Hua W., Liu Y.-L., Zhou Y.-L., Luo H.-W., Jiang F.-N., Zhong W.-D. (2015). Dysregulated microRNA-224/apelin axis associated with aggressive progression and poor prognosis in patients with prostate cancer. Hum. Pathol..

[214] Zhou Y., Zhao M., Du Y., Liu Y., Zhao G., Ye L., Li Q., Li H., Wang X., Liu X. (2019). MicroRNA-195 suppresses the progression of lung adenocarcinoma by directly targeting apelin. Thorac. Cancer.

[215] Liao Y.-C., Wang Y.-S., Hsi E., Chang M.-H., You Y.-Z., Juo S.-H.H. (2015). MicroRNA-765 influences arterial stiffness through modulating apelin expression. Mol. Cell. Endocrinol..

[216] Yang M., Song J.-J., Yang X.-C., Zhong G.-Z., Zhong J.-C. (2022). MiRNA-122-5p inhibitor abolishes angiotensin II–mediated loss of autophagy and promotion of apoptosis in rat cardiofibroblasts by modulation of the apelin-AMPK-mTOR signaling. In Vitro Cell. Dev. Biol. Anim..

